# Interpretation and identification of within-unit and cross-sectional variation in panel data models

**DOI:** 10.1371/journal.pone.0231349

**Published:** 2020-04-21

**Authors:** Jonathan Kropko, Robert Kubinec

**Affiliations:** 1 School of Data Science, University of Virginia, Charlottesville, Virginia, United States of America; 2 Division of Social Sciences, New York University Abu Dhabi, Abu Dhabi, United Arab Emirates; University of Glasgow, UNITED KINGDOM

## Abstract

While fixed effects (FE) models are often employed to address potential omitted variables, we argue that these models’ real utility is in isolating a particular dimension of variance from panel data for analysis. In addition, we show through novel mathematical decomposition and simulation that only one-way FE models cleanly capture either the over-time or cross-sectional dimensions in panel data, while the two-way FE model unhelpfully combines within-unit and cross-sectional variation in a way that produces un-interpretable answers. In fact, as we show in this paper, if we begin with the interpretation that many researchers wrongly assign to the two-way FE model—that it represents a single estimate of X on Y while accounting for unit-level heterogeneity and time shocks—the two-way FE specification is statistically unidentified, a fact that statistical software packages like R and Stata obscure through internal matrix processing.

## Introduction

In designing a statistical model, applied researchers take steps to help the model achieve unbiasedness, consistency, and efficiency. But an even more important goal is for the model to be *useful*. For a model to be useful it must have an interpretation that provides a clear answer to the research question posed. In this paper, we show that the commonly applied two-way fixed model (two-way FE) only has such an interpretation in the difference-in-differences (DiD) causal framework. However, many if not most empirical applications involve data that has a structure that does not meets the stringent criteria of the DiD framework. So what is the appropriate interpretation to assign to the two-way FE model coefficient if it cannot be understood as the DiD treatment effect?

A DiD estimate provides a causal effect if certain assumptions are met, such as the parallel paths assumption [1]. DiD also requires a particular structure for the data being analyzed: two time periods, denoted as the pre and post-treatment times, and a treatment variable that is 0 for for the non-treated cases and for all cases during the pre-treatment and 1 for the treated cases during the post-treatment [2, 3]. We define a case to be the unit of analysis in the data that is observed at repeated points in time. For example, cases may be countries, individual respondents in a longitudinal survey, elected officials, and so on. If the data conform to these specifications, then the two-way FE model’s coefficient is an unbiased and efficient estimate of the DiD statistic [4]. However, if the data include more than two time periods, then the two-way FE model’s estimand is not the same as the DiD statistic. As such, the two-way FE model should not be thought of as a generalized form of a DiD design when there are more than two time periods in the data, as is often tacitly assumed.

While many recent studies advance our methodological toolkit with regard to better understanding DiD as a framework for panel data modeling [5, 6], we do not offer in this paper a new methodology for DiD estimation. Rather, our aim is to describe how the two-way FE model can be interpreted and understood in plain language that is relevant to the descriptive and exploratory analyses for which panel data is often used. While causal inference is very important, we note that it is often necessary with panel data to estimate models in which strict causal identification assumptions are implausible but that nonetheless answer important questions of interest to the research community. In this paper we want to provide researchers with the tools they need to thoroughly understand the extraordinary resource of the many panel data sets created in the last three decades. We believe that researchers already have a potent method of analysis for panel data: the standard linear model with fixed effects on time points or cases (but not both). Widely employed, but as we show, poorly understood, this simple specification offers great promise to unlock insights in the data we have.

We re-analyze fixed effects (FE) models from this perspective and argue that their merit is in capturing distinct dimensions of variation in time-series cross-sectional (TSCS) datasets. By understanding exactly what FE models do and do not represent in terms of the observed variation, researchers can employ them confidently and explore empirical results with much more detail than is commonly done. Establishing what utility FE models have should also help resolve ongoing debates on how to best represent heterogeneity in effects over time (autocorrelation) and heterogeneity in effects over space (spatial autocorrelation).

In this paper we mathematically decompose the one-way and two-way FE models in a way that shows clearly how results from these models should be understood and interpreted. Including a second set of FEs dramatically changes the quantity estimated and, in a majority of cases, no longer answers the research question posed. While researchers turn to the two-way FE model because it supposedly accounts for more omitted variables than a one-way model, we show that it does so at the cost of the model’s interpretability and usefulness. While our analysis of the one-way FE model follows the existing literature, our exposition of the two-way FE model is novel. We are also the first to show both formally and with simulations that if we begin with the assumptions that many researchers bring to the two-way FE model—that it represents a single estimate of X on Y while accounting for unit-level heterogeneity and time shocks—this specification is in fact statistically unidentified. This concerning fact about two-way FE models has probably remained hidden for so long because statistical software packages like R and Stata employ hidden matrix processing to work around the unidentifiability.

We urge researchers to choose between models by taking into consideration the way in which parameters must be interpreted. A correct answer to the wrong question is as useless as the wrong answer to the right question. There is little to be gained by applying models that result in answers that are irrelevant to the fundamental question at hand, and there are well-known strategies to address omitted variable bias that do not result in uninterpretable models, such as including relevant control variables and employing research designs that can explicitly draw the comparisons of interest in the data. If such strategies are unavailable or do not work, then researchers should present the correct model and provide warnings about issues they were unable to address rather than employ a model that cannot be understandably interpreted. Because of the restrictive assumptions and difficulty in substantive interpretation of the two-way FE model, we do not recommend that applied researchers rely on this model except for situations in which the model’s interpretation exactly matches the researcher’s intended research question and the model’s assumptions are taken into account.

## The use of one-way and two-way FEs in applied research

**Fig 1:**
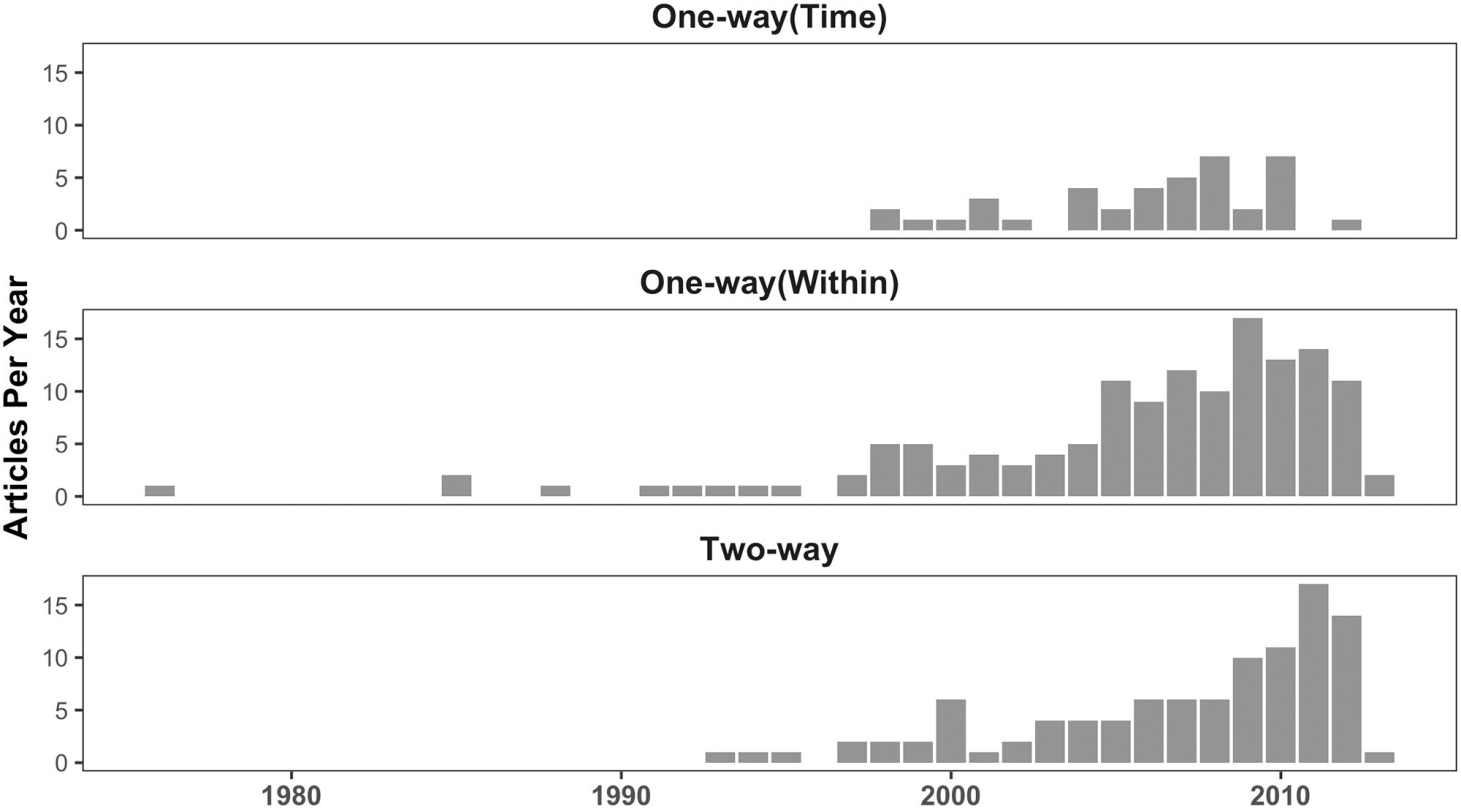
Fixed effects models in political science over time.

Our reason for focusing on fixed effects models is because these are some of the most popular estimation tools in political science. To document this, we coded papers in the general interest journals in political science—*The American Political Science Review*, *The American Journal of Political Science*, and *The Journal of Politics*—dating back to 1976. We coded each paper that used the term “fixed effect” for whether the model employed one-way FEs on cases, one-way FEs on time points, or two-way FEs. The results of this analysis are shown in Fig 1. Fixed effects models are used in a truly astonishing number of papers even considering only these three journals.

**Fig 2:**
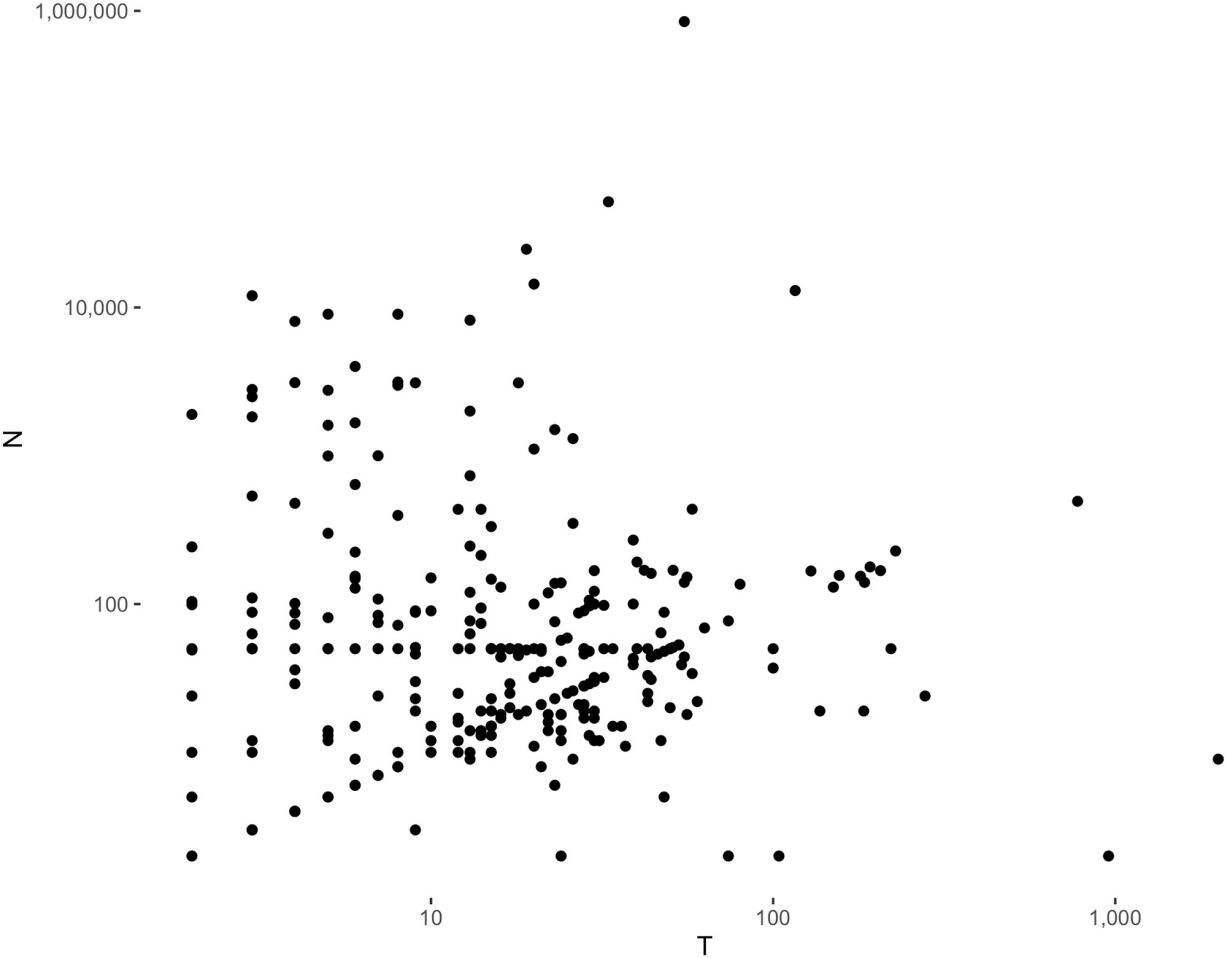
Number of cases (N) and time points (T) in political science panel data papers, 1976–2014.

The additional trend that is apparent from this figure is the growing share of two-way FE models over other FE models. The growing dominance of this model is what motivated this work as the review of papers showed there is no universally agreed-upon interpretation of this model in terms of the underlying dimensions of variation in the data. Researchers usually report the two-way FE model or a two-way and one-way FE model without much consideration to their differences and with the assertion that the two-way FE represents the robust effect of X on Y. Given how strongly political scientists rely on these models, we believe that a thorough re-assessment of these models would benefit applied research in the discipline. As Fig 2 shows, most of the papers published in the top 3 journals in the last three decades have had more than two cases and two time points, rendering them outside of the standard DiD framework.

Before we proceed further, we define the important models and terms under consideration in this paper. We begin with a review of notation for FE models. All of the FE models we describe can be estimated with classical OLS by including indicators for cases, time points, or both in the model along with the independent variables. First we define the two versions of the one-way FE models: the case FE model and the time FE model. The case FE model with one regressor is

$$\begin{array}{c} y_{it}=\alpha_{i}+\beta x_{it}+\varepsilon_{it}, \end{array}$$ (1)

where *i* denotes cases and *t* denotes time points, *α*_*i*_ represents case-specific intercepts, the *β* coefficient is fixed across cases, and *ε*_*it*_ represents residuals. The model may include a global intercept while excluding one case-specific intercept to avoid collinearity. Similarly, the time FE model with one regressor is

$$\begin{array}{c} y_{it}=\alpha_{t}+\beta x_{it}+\varepsilon_{it}, \end{array}$$ (2)

where *α*_*t*_ represents time-specific intercepts, and the two-way FE model is

$$\begin{array}{c} y_{it}=\alpha_{i}+\alpha_{t}+\beta x_{it}+\varepsilon_{it}. \end{array}$$ (3)

These models can be considered special cases of multilevel or hierarchical models in which the case or time-specific intercepts are assumed to have improper uniform prior distributions, or equivalently normal prior distributions with infinite variance. [7] refer to such models as “no pooling” because the case or time-specific intercepts are estimated using only the information within individual cases or time points [7]. If instead of no pooling, intercepts are estimated using partial pooling by calculating a weighted average of each individual intercept with the mean across these intercepts [7], then the resulting model is what is referred to in econometrics as a random effects (RE) model. FE models are generally preferred in econometrics and political science to RE models because of concerns that the intercepts, considered part of the residual in RE models, could be endogenous to the regressors in a linear model [8], although increasingly multilevel models are also being employed in these disciplines.

Unfortunately, the phrases “fixed effect” and “random effect” take on different meanings in different disciplines, leading to some confusion that we would like to avoid. In multilevel modeling “random effect” refers to coefficients that vary by group and “fixed effect” refers to coefficients that are fixed across groups [7]. In this paper we follow the conventions in econometrics and define “fixed effect” to refer to a model in which case or time-specific intercepts are estimated via OLS with no informative prior distribution, and “random effect” to refer to the partial pooling model. Going forward, our focus is on fixed effect models, though the interpretation of intercepts as representing dimensions of variance is still useful for researchers employing multilevel models.

Many authors have come to prefer FE models for their ability to remove potential confounders [8]. This approach was the justification for the well-known “Dirty Pool” criticism leveled by [9]: “the problem of ignoring fixed effects is a special case of a more general problem, that of omitting variables in multivariate regression” (443). This rationale sends a strong message to researchers that they must either include fixed effects or accept a biased coefficient. During our literature review, we encountered several papers in which the author wanted to use a FE model, but instead employed a different TSCS model because the FE model failed to produce estimates, usually because of a lack of variation in the variable of interest. As a consequence, these authors believe that a model *which could not fit their data* must in fact be the right model, a sign that incorrect interpretations of these models are having a serious effect on research practice. To take one example, [10] does not use a one-way case FE model because “fixed effects leads to too many observations dropping from the analysis” (709). In Donno’s case, the independent variable of interest is electoral system, a variable which will only rarely change over time within countries. As a result, the inclusion of case FEs results in all countries dropping from the model that did not experience at least one change in electoral system, such as the United States. As we explain elsewhere in this paper, the problem is not that variation in electoral systems does not exist, but rather that it mostly does not exist within cases, which is the dimension of variation on which the case FE estimator operates. This preference for case FEs, even in situations when the variation appears more appropriate for time FEs, is what we argue explains the small number of time FE models in published work, as illustrated by the narrow region representing one-way (time) FE models in Fig 1.

Increasingly, scholars are revising our understanding of fixed effects papers across disciplines. Two recent papers are of particular importance in this regard. First, [11] show that the assumptions researchers use when evaluating fixed effects models do not in fact correspond to their research questions of interest. Second, [12] use a decomposition of fixed effects models in terms of weights that allows them to re-construct the estimator as a weighted average, permitting non-parametric inference. Both of these recent articles suggest that there is growing momentum for a revision of how fixed effects models are employed in analytic research.

In one sign of this trend, scholars have begun to examine FE models in terms of the potential outcomes framework [12, 13], which allows researchers to avoid assuming that a single linear fixed effects specification must be correct. At the same time, the potential outcomes framework is limited in that it cannot decide which potential outcomes are most relevant to a research question. As such, even though our research does not emphasize the potential outcomes framework, we do believe that the questions we raise for interpretation are relevant for researchers working in this field as it is still necessary to interpret average treatment effects in terms of the dimensions of variance within the data.

Many scholars have employed the two-way FE model as a form of DiD [14–18]. Two-way FE models, as we explain in the S3 Appendix, represent a particular combination of case and time variation that returns a DiD estimate under this canonical design, but does not estimate the DiD in more general contexts unless very strong model-based assumptions are used. Furthermore, two-way FEs are not the only way that researchers can estimate a DiD. The standard DiD design can also be estimated by either a difference of means t-test or a one-way time FE model depending on how the treatment variable is coded in the dataset [2]. This discrepancy between what people say the two-way fixed effects model is, and what it in fact is, is what motivates extensions of the DiD framework that can incorporate multi-period panel data [12].

Other than commenting on the relationship between the FE model and difference-in-difference, we do not further analyze the relationship between the FE model and potential outcomes. While this is a fruitful area for exploration, our intention is to guide the majority of researchers who primarily employ FE models with observational data where causal identification can rarely be assumed. At the same time, we believe our re-interpretation of FE models is helpful for researchers working in the potential outcomes framework as any estimator must select exactly which of the *N* × *T* potential outcomes in the dataset are relevant to a research question. For example, [12] develop a within-unit matching estimator which provides a non-parametric estimation of a one-way FE model for cases, but as we show, that is only one of several possible questions that can be asked about a TSCS dataset. Regardless of what assumptions the researcher brings to a TSCS dataset, she must be aware of the two primary dimensions of variation: over-time *and* cross-sectional dimensions. As Fig 2 shows, there is in fact more variation among political science panel data in the cross-section (i.e., the size of N), but most fixed effects models instead emphasize over-time variation, potentially impoverishing the empirical results that can be obtained.

## Interpretation of FE models

### Interpretation of one-way FE coefficients

We begin by reviewing existing derivations of the one-way FE model so that we have a framework in which to compare the one-way and two-way FE models. There are several ways to implement fixed effects that are equivalent to including dummy variables for cases or for time points. The one-way case FE estimator can be derived by subtracting the mean across observations within each case (see [19]; [20]; and [8]). In other words, we transform the outcome as follows:

$$\begin{array}{c} y_{it}^{*}=y_{it}-{\bar{y}}_{i}=y_{it}-\frac{1}{|T_{i}|}\sum_{t\in T_{i}}y_{it}. \end{array}$$ (4)

Here *T*_*i*_ is the set of time points observed for case *i* and |*T*_*i*_| is the number of time points in this set. This model removes all possible covariates that vary across cases but are fixed across time, regardless of whether or not those covariates are observed. Consider for example an outcome given by a linear model

$$\begin{array}{c} y_{it}=\alpha +\beta x_{it}+\delta u_{i}+\varepsilon_{it} \end{array}$$ (5)

that includes an independent variable *x*_*it*_ that varies both across cases and time points, and an independent variable *u*_*i*_ that varies across cases but is fixed across time. If we apply the transformation in Eq 4 to this linear equation, the result is

$$\begin{array}{cc} y_{it}-\frac{1}{|T_{i}|}\sum_{t\in T_{i}}y_{it} & =(\alpha +\beta x_{it}+\delta u_{i}+\varepsilon_{it})-\frac{1}{|T_{i}|}\sum_{t\in T_{i}}(\alpha +\beta x_{it}+\delta u_{i}+\varepsilon_{it})\\ & =\beta (x_{it}-\frac{1}{|T_{i}|}\sum_{t\in T_{i}}x_{it})+(\varepsilon_{it}-\frac{1}{|T_{i}|}\sum_{t\in T_{i}}\varepsilon_{it}). \end{array}$$

Importantly, the time-fixed covariate *u*_*i*_ drops out of the model. The coefficient *β* in Eq 5 is then estimated by OLS to be

$$\begin{array}{c} {\hat{\beta }}_{\text{caseFE}}=\frac{\sum_{i=1}^{N}\sum_{t=1}^{T_{i}}\left(x_{it}-{\bar{x}}_{i}\right)\left(y_{it}-{\bar{y}}_{i}\right)}{\sum_{i=1}^{N}\sum_{t=1}^{T_{i}}{\left(x_{it}-{\bar{x}}_{i}\right)}^{2}}. \end{array}$$ (6)

Likewise, the one-way time FE estimator subtracts the mean across observations within each time point,

$$\begin{array}{c} y_{it}^{*}=y_{it}-{\bar{y}}_{t}=y_{it}-\frac{1}{|N_{t}|}\sum_{i\in N_{t}}y_{it} \end{array}$$ (7)

where *N*_*t*_ is the set of cases observed at time point *t* and |*N*_*t*_| is the number of cases in this set. Unlike the case FE model, the time FE model cannot eliminate a time-fixed covariate *u*_*i*_; however it does eliminate variables that vary over time but are fixed across cases. The OLS estimate of a coefficient *β* on a covariate *x*_*it*_ in a time FE model is

$$\begin{array}{c} {\hat{\beta }}_{\text{timeFE}}=\frac{\sum_{t=1}^{T}\sum_{i=1}^{N_{t}}\left(x_{it}-{\bar{x}}_{t}\right)\left(y_{it}-{\bar{y}}_{t}\right)}{\sum_{t=1}^{T}\sum_{i=1}^{N_{t}}{\left(x_{it}-{\bar{x}}_{t}\right)}^{2}}. \end{array}$$ (8)

Going forward, we refer to this operationalization of fixed effects as the *mean-centering* approach.

Another way to implement fixed effects is the *data subsetting* approach. Case FEs only work with the time series—not the cross-sections—in the data because a coefficient from this model is a weighted average of the coefficients we obtain by subsetting the data by case. To demonstrate this point, note that we can multiply and divide a factor of $\sum_{t=1}^{T_{i}}{\left(x_{it}-{\bar{x}}_{i}\right)}^{2}$ within the summation across cases in the numerator in Eq 6,

$$\begin{array}{c} {\hat{\beta }}_{\text{caseFE}}=\frac{\sum_{i=1}^{N}\sum_{t=1}^{T_{i}}{\left(x_{it}-{\bar{x}}_{i}\right)}^{2}\frac{\sum_{t=1}^{T_{i}}\left(x_{it}-{\bar{x}}_{i}\right)\left(y_{it}-{\bar{y}}_{i}\right)}{\sum_{t=1}^{T_{i}}{\left(x_{it}-{\bar{x}}_{i}\right)}^{2}}}{\sum_{i=1}^{N}\sum_{t=1}^{T_{i}}{\left(x_{it}-{\bar{x}}_{i}\right)}^{2}}, \end{array}$$ (9)

which can be rewritten as

$$\begin{array}{c} {\hat{\beta }}_{\text{caseFE}}=\frac{\sum_{i=1}^{N}\omega_{i}{\hat{\beta }}_{i}}{\sum_{i=1}^{N}\omega_{i}} \end{array}$$ (10)

where ${\hat{\beta }}_{i}$ is the OLS coefficient estimated using only the data within one case, and

$$\begin{array}{c} \omega_{i}=\sum_{t=1}^{T_{i}}{\left(x_{it}-{\bar{x}}_{i}\right)}^{2}=T_{i}\times V\left(x_{it}\right). \end{array}$$ (11)

This result implies that running a case FE model is equivalent to taking these steps:

1. Consider only the observations for case 1. Since all of these observations come from the same case, all of the variation exists over time.
2. Regress *y*_1*t*_ on *x*_1*t*_ using this subset of the data, and record the coefficient *β*_1_.
3. Calculate the variance of the values of *x* for case 1 and the sample size within this subset *T*_1_, and record the product *ω*_1_ = *T*_1_ × *V*(*x*_1*t*_).
4. Repeat steps 1, 2, and 3 for every case in the data.
5. Calculate the case FE coefficient using Eq 10 by taking the average of every case-specific coefficient *β*_*i*_, weighted by *ω*_*i*_.

Likewise, the time FE estimator produces a coefficient estimate that is a weighted (by variance and sample size) average of the coefficients we calculate for each cross-section. We present this argument again as a formal mathematical proof in S3 Appendix in the supplemental material.

The data subsetting approach to operationalizing fixed effects leads to a clear interpretation of one-way FE coefficients. Within the data for one case all variation must occur over time, so a regression coefficient within this subset must be interpreted as the average effect of a unit-increase in *x* on *y* as each variable changes over time for this specific case. Because case FE coefficients average these corresponding coefficients across all cases, a case FE coefficient represents the average effect of a unit-increase in *x* on *y* as each variable changes over time, generalized to all cases. Similarly, within one time point in the data all variation is cross-sectional, so a regression coefficient within this subset must be interpreted as the average effect of a unit-increase in *x* on *y* as each variable changes from case to case at this specific point in time. Therefore time FE coefficients represent the average effect of a unit-increase in *x* on *y* as each variable changes from case to case, generalized across all time points.

For example, with regard to the question of whether economic development in a country affects the quality of that country’s democracy, we can investigate individual countries over time or particular cross-sections of countries in a specific year. If we look only at the time series for India, we ask “as GDP increases for India over time, how does the quality of its democracy change over time?” If we use case FEs, we generalize this question across countries: “as GDP increases for *a country* over time, how does the quality of its democracy change over time?” If instead we look at the cross-section that exists in 1990, we ask “how much more democratic are wealthier countries than poorer countries in 1990?” Time FEs generalize this question to the entire time frame under analysis, and simply ask “how much more democratic are wealthier countries than poorer countries at any point in time?” If a researcher intends to compare one case to itself over time, it is appropriate to examine individual time series and to use case FEs; if a researcher intends to compare one case to another at the same point in time, it is appropriate to examine cross-sections and to use time FEs. If a researcher wishes to allow different cases to experience different over-time effects, or to let different cross-sectional effects exist at different points in time, it is straightforward to use interactions to extend a one-way FE specification to account for the desired heterogeneity.

Employing a one-way FE model in a way that can answer the research question, on the other hand, does not guarantee that it will do so. That is, selecting a model with a correct interpretation is a necessary but not a sufficient condition for successful statistical analysis. Indeed, time series in TSCS data may have all of the well known problems of time series in non-panel contexts: seasonality, non-stationarity, stochastic volatility, and so on. Likewise, cross-sections in TSCS data may exhibit reverse causality, heteroskedasticity, multicollinearity, etc. Both time series and cross-sections can also suffer from omitted variable bias if there are unmeasured confounders along the dimension of variance in the model. While these issues must be addressed, they must be addressed in a way that still allows researchers to interpret the model.

### Confoundedness and the FE model

As the previous section shows, FE models can remove time-invariant omitted variables, case-invariant omitted variables, or both from the variance of an outcome. Indeed, FE models are usually chosen and justified for this reason. Here, before moving on to our derivation of the two-way FE model, we want to address this property of FE models. We argue that the ability of an FE model to remove these confounders is a *side effect* of the fact that FEs isolate particular dimensions of variance in the data to analyze. If a model only looks at variation over time, then logically, no variable that is fixed over time may impact the outcome. Likewise, no variable that is fixed across cases can explain the cross-sectional differences in the data.

As such, while it is correct to state that FE models remove time-invariant and/or case-invariant confounders, it is not correct to say that FE models *control* for confounding variables. While this distinction is subtle, it is an important one to make as applied research often uses these terms interchangeably. For a given confounding variable *z*_*it*_ that is correlated with the effect of interest *x*_*it*_, controlling for *z*_*it*_ involves obtaining a set of measurements of *z*_*it*_ (i.e., data) and including those in the regression model with *x*_*it*_. Doing so will produce a *β* for *x*_*it*_ that is estimated *marginal* of the *β* for *z*_*it*_, implicitly averaging over the effect of *z*_*it*_ on *y*_*it*_ and blocking the “back-door” path from *x*_*it*_ to *y*_*it*_.

However, as the analysis makes clear in the previous section, this kind of marginalization is *not* what is occurring in a fixed effects model by either including dummy variables or de-trending. Rather, a transformation is being applied to *x*_*it*_ such that the *β* represents a distinct dimension of variation of *x*_*it*_. In other words, the *β* from the fixed effects model, while it is no longer affected by the relationship between *z*_*it*_ and *x*_*it*_, *is not the same as the β* for *x*_*it*_ that would have been attained by including a measured covariate for *z*_*it*_ *in a regression model*. This is the reason we are emphasizing issues of interpretation in this paper rather than the ever-present threat of lurking omitted variables. Whether or not the fixed effects *β* is preferable to a naive *β* for *x*_*it*_ is not a question primarily of bias as fixed effects themselves do not control for any specific variable. Instead, the first priority in choosing a panel data model should be in identifying one that can answer the research question posed, with threats to inference a vitally important but nonetheless secondary criteria.

### Interpretation of two-way FE coefficients

We show in this section why the two-way FE model is in a fact a radical departure from the one-way FE models and produces an often un-interpretable estimate. As with one-way FE models, we can express the two-way FE model by using a mean-centering or a data subsetting approach to operationalizing the fixed effects along each dimension. First, we mean-center the variables within both cases and time points by subtracting the mean along one dimension, then subtracting the mean of these differences along the other dimension. If the panels in the data are balanced—exactly the same time points are observed for every case—then [20] and others write the two-way FE coefficient estimate that we denote ${\hat{\beta }}_{TW}$ as

$$\begin{array}{c} {\hat{\beta }}_{TW}=\frac{\sum_{i=1}^{N}\sum_{t=1}^{T}\left(y_{it}-{\bar{y}}_{i}-{\bar{y}}_{t}+\bar{y}\right)\left(x_{it}-{\bar{x}}_{i}-{\bar{x}}_{t}+\bar{x}\right)}{\sum_{i=1}^{N}\sum_{t=1}^{T}{\left(x_{it}-{\bar{x}}_{i}-{\bar{x}}_{t}+\bar{x}\right)}^{2}}. \end{array}$$ (12)

Covariates that are fixed across time are removed from the model through the subtraction of the case-means of each variable. Simultaneously, covariates that are fixed across cases are removed through the subtraction of the time-means.

However, the most useful interpretation of coefficients from the two-way FE model is revealed when we apply mean-centering to one dimension and data subsetting on the other. It is not possible to use data subsetting on both cases and time points, because by definition in TSCS data there is only one observation per case and time, which is insufficient to identify a regression coefficient. Without loss of generality, we mean-center with regard to cases and we subset with regard to the time points. Given *N* cases, *T* time points, and data *y*_*it*_ and *x*_*it*_, mean-centering requires transforming the variables and residuals as follows:

$$\begin{array}{c} \begin{array}{ccccc} y_{it}^{*}=y_{it}-{\bar{y}}_{i}, & & x_{it}^{*}=x_{it}-{\bar{x}}_{i}, & & \varepsilon_{it}^{*}=\varepsilon_{it}-{\bar{\varepsilon }}_{i}. \end{array} \end{array}$$

Then, to apply data subsetting on time points, we consider each time point *t* ∈ {1,…,*T*} individually and for each we calculate a coefficient *β*_*t*_ from the equation

$$\begin{array}{c} y_{it}^{*}=\alpha_{t}+\beta_{t}x_{it}^{*}+\varepsilon_{it}^{*}. \end{array}$$ (13)

A two-way FE coefficient has an interpretation that generalizes the interpretation of *β*_*t*_ in Eq 13 across time. So, to understand what a two-way FE coefficient means, we must understand what *β*_*t*_ means. Equivalently, we can mean-center with regard to time points and apply data subsetting on cases: then a two-way FE coefficient has an interpretation that generalizes the interpretation of *β*_*i*_ in

$$\begin{array}{c} y_{it}^{*}=\alpha_{i}+\beta_{i}x_{it}^{*}+\varepsilon_{it}^{*}, \end{array}$$

where $y_{it}^{*}=y_{it}-{\bar{y}}_{t}$, $x_{it}^{*}=x_{it}-{\bar{x}}_{t}$, and $\varepsilon_{it}^{*}=\varepsilon_{it}-{\bar{\varepsilon }}_{t}$.

**Fig 3:**
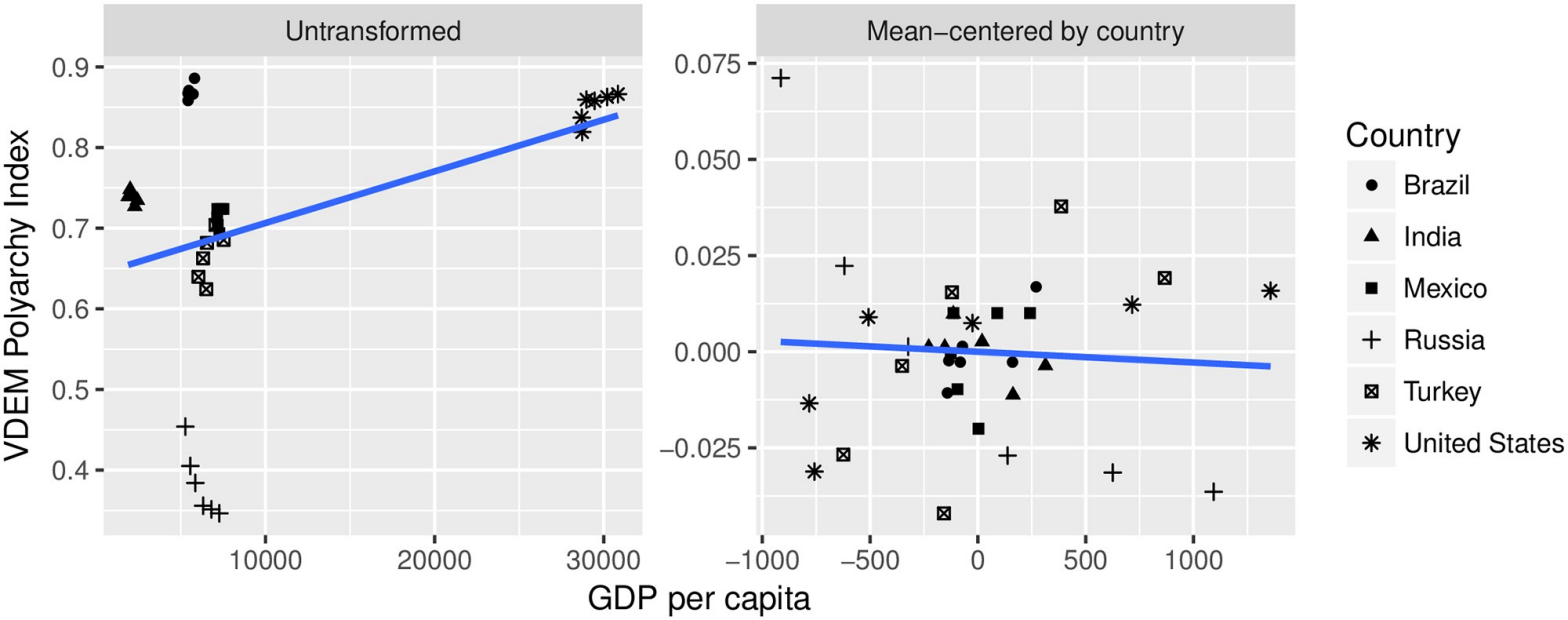
GDP and democracy data from the varieties of democracy dataset, 2000–2005.

For clarity, we use real data to illustrate the proper interpretation of the two-way FE coefficient. We return to the example of GDP and democracy: we employ the Varieties of Democracy (VDEM) polyarchy index for the quality of a country’s democracy, and the measure of GDP per capita included in the VDEM data [21]. We keep six countries—Brazil, India, Mexico, Russia, Turkey, and the United States—and the six years from 2000 through 2005. Fig 3 includes two panels. The left-hand panel is a scatterplot of each variable, untransformed. The slope of the best-fit line in the left-hand panel is the pooled OLS coefficient. In the right-hand panel, the country-specific means have been subtracted from both democracy and per capita GDP. The slope of the best-fit line in the right-hand panel is the coefficient from the case FE model.

**Fig 4:**
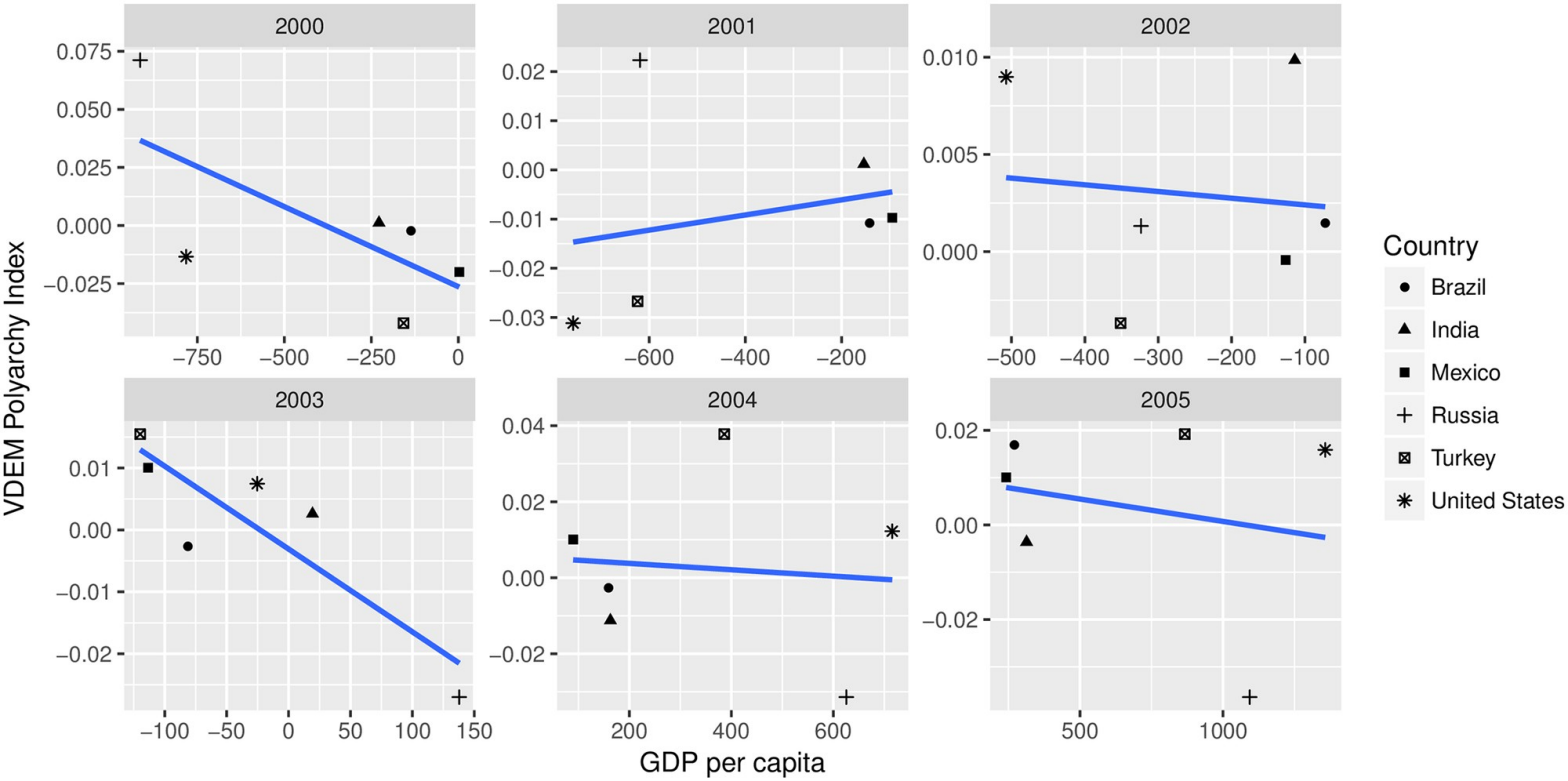
Subsetting the country-mean centered data by year.

To proceed from the case FE coefficient to the two-way FE coefficient, we subset the data in the right-hand panel of Fig 3 by year. These six scatterplots are displayed in Fig 4.

Each best-fit line in each panel represents another entry for *β*_*t*_ in Eq 13. The two-way FE coefficient is an average of these slopes, weighted by the amount of data in each scatterplot times the variance of the *x*-values in each plot. But to understand the substantive meaning of the two-way FE coefficient, it is necessary to describe the substantive meaning of the slope in each of the plots in Fig 4. Consider the plot for the year 2000. The *x*-axis represents how, in the year 2000, a single country’s GDP per capita compares to that country’s mean GDP per capita from 2000 to 2005. Likewise, the *y*-axis represents how, in the year 2000, a single country’s democracy index compares to that country’s mean democracy index from 2000 to 2005. The negative slope in 2000 means that, on average, relative to a country with a GDP per capita that is farther below its own over-time mean, another country with a GDP per capita that is closer to *its* over-time mean will have a democracy index that is farther below that other country’s democracy index’s over-time mean. The two-way FE coefficient generalizes this interpretation to all years between 2000 and 2005 by calculating the weighted average of the six slopes that appear in Fig 4. The previous two sentences represent our best effort to provide an intuitive expression of ${\hat{\beta }}_{TW}$ in Eq 12.

This interpretation, or the equivalent one that mean-centers by year and subsets by country, will often be difficult to communicate and to understand. It is a common mistake to interpret two-way FE coefficients as if they draw the same comparison in the data that case FE coefficients speak to, when in fact they speak to a more complex comparison. Even if the two-way FE coefficients are interpreted correctly, this interpretation may not match the question the model is intended to answer. In that case, we suggest that applied researchers employ methods with interpretations that directly answer the research question.

This interpretation of two-way FE coefficients means that the two-way FE model is a complex amalgamation of cross-sectional and temporal effects in TSCS data.

As a result, although the two-way FE estimator removes case-fixed and time-fixed omitted variables, it does not isolate either the variation across cases or the variation across time in TSCS data. Thus, if researchers have the goal of removing problematic variation from the dependent variable, then the two-way FE estimate paradoxically accomplishes the opposite of what these researchers intend. While the cross-sectional variance is removed from case FEs this variance is present for time FEs, so it must be present in the two-way FE model as well. Likewise, while the temporal variation is omitted from time FEs it exists in case FEs, so it is present in two-way FEs.

## Simulating TSCS data with known within-case and within-time slopes

The correct interpretation of two-way FE coefficients, as described in the previous section, makes it clear that the two-way FE model combines over-time and cross-sectional variance in a way is difficult to understand in substantive terms. In this section, we use simulations to illustrate the relationship between the two-way FE coefficient and the slopes that exist within subsets of the data by time point and by case, slopes which the time FE and case FE models can return. In that way we can gain a better understanding of the substantive meaning of the two-way FE coefficient.

We want to emphasize that our use of a simulation here departs from the purpose simulations commonly serve in methods papers. We are not trying to demonstrate any point about bias or coverage of FE models estimated with OLS. We are instead trying to shed light on the relationship between one and two-way FE models to help researchers understand the implications of including the second set of FEs.

To conduct this simulation, we need a way to generate data that allows us to set the slopes within case-specific subsets of the data and within time-specific subsets of the data at the same time. To that end, there are two reasons why we cannot use a standard approach to generating TSCS data. First, most prior work that we are aware of adopts one of the fixed effects models and draws the fixed effects and a covariate from a uniform or normal distribution along with normally-distributed errors and a fixed *β*, such as in the following:

$$\begin{array}{c} \begin{array}{cc} \epsilon_{it} & ∼N(0,1)\\ \alpha_{i} & ∼U(-2,2)\\ \alpha_{t} & ∼U(-2,2)\\ X_{it} & ∼U(-2,2)\\ \beta & =3\\ y_{it} & =\alpha_{i}+\alpha_{t}+\beta x_{it}+\epsilon_{it} \end{array} \end{array}$$

If this data is then estimated with a linear regression model, the resulting coefficient of *β* will be unbiased around 3.

This straightforward simulation can be used to justify the two-way FE model, but if the data are generated from a two-way FE equation then it is tautological that two-way FE outperforms competitors in modeling the data. As a result, we cannot understand the properties of the two-way FE model by using data generated from the two-way FE linear equation. Second, it is impossible to set both the within-time and within-case slopes in a single equation. It is possible, however, to set both slopes using a simple system of two equations, which is how we proceed.

To express the within-time slopes, we use a simple bivariate regression model that implies the following expected value for *y*_*it*_:

$$\begin{array}{c} E\left(y_{it}\right)=\alpha_{t}+\beta_{t}x_{it}. \end{array}$$ (14)

In this model each cross-section has its own intercept *α*_*t*_ and its own coefficient, or within-time slope, on *x*_*it*_, which is *β*_*t*_. If the coefficients on *x*_*it*_ within each cross-section are all the same, then *β*_*t*_ = *β*, ∀*t*, which corresponds to a standard one-way FE regression with fixed effects on time points.

To express the within-case slopes, we use another simple bivariate regression model that implies the following expected value for *y*_*it*_:

$$\begin{array}{c} E\left(y_{it}\right)=\alpha_{i}+\gamma_{i}x_{it}. \end{array}$$ (15)

As with Eq 14, the regressions in each time series may have unique intercepts, *α*_*i*_, and within-case slopes, *γ*_*i*_, and if *γ*_*i*_ = *γ*, ∀*i*, then we have a standard one-way FE regression with fixed effects on cases. Since Eqs 14 and 15 describe the same data while focusing on different dimensions within those data, both equations can simultaneously be true for a given TSCS dataset. To accomplish this task, we generate data from both Eqs 14 and 15 simultaneously. Accordingly, we set up a system of simultaneous equations

$$\begin{array}{c} \left\{ \begin{array}{c} E\left(y_{it}\right)=\alpha_{t}+\beta_{t}x_{it},\\ E\left(y_{it}\right)=\alpha_{i}+\gamma_{i}x_{it}, \end{array}\right. \end{array}$$ (16)

and solve it for *E*(*y*_*it*_) and *x*_*it*_ (see S3 Appendix in the supplemental material for the derivation of this solution):

$$\begin{array}{c} x_{it}=\frac{\alpha_{i}-\alpha_{t}}{\beta_{t}-\gamma_{i}} \end{array}$$ (17)

and

$$\begin{array}{c} E\left(y_{it}\right)=\frac{\beta_{t}\alpha_{i}-\gamma_{i}\alpha_{t}}{\beta_{t}-\gamma_{i}}. \end{array}$$ (18)

To include a stochastic component in the dependent variable, we add an exogenous error term to *E*(*y*_*it*_) to generate the outcome,

$$\begin{array}{c} y_{it}=\frac{\beta_{t}\alpha_{i}-\gamma_{i}\alpha_{t}}{\beta_{t}-\gamma_{i}}+\varepsilon_{it}. \end{array}$$ (19)

In the following simulations, we generate data that corresponds both to Eqs 14 and 15 by generating *x*_*it*_ from Eq 17 and *y*_*it*_ from Eq 19.

**Fig 5:**
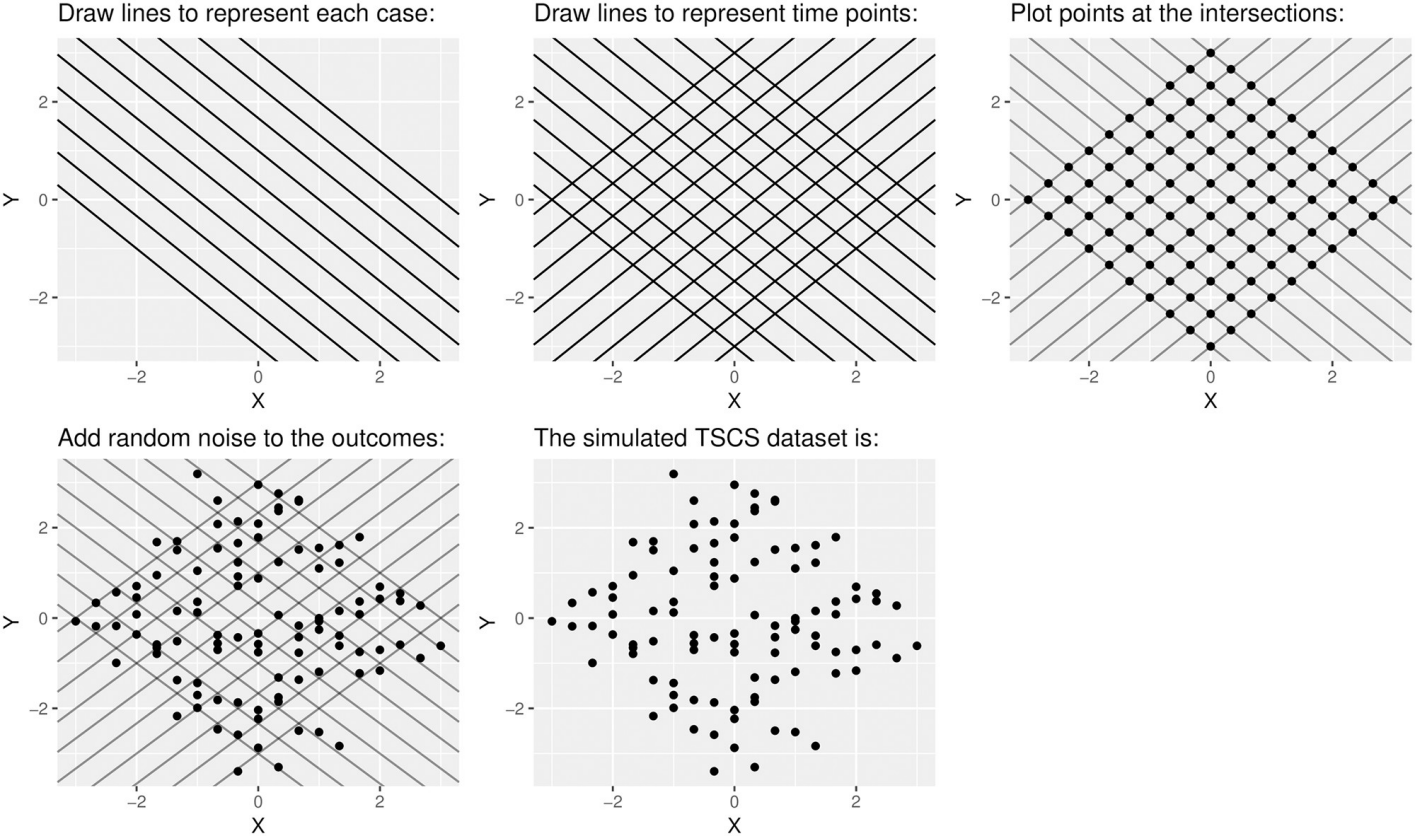
Example of the generation of one TSCS dataset.

While our simulation differs from the common method of generating data from either a linear model with one-way or two-way FE, we maintain that it is the simplest method for simulating TSCS data in which we can control both the within-case and within-time slopes in the data. To more clearly demonstrate the logic of our approach, we next provide an example of our simulation in which we simulate 10 cases and 10 time points for 100 total observations. We set the case-specific intercepts and the time-specific intercepts to values from -3 to 3 in increments of 0.6. We set the within-case slopes to each be -1, and the within-time slopes to each be 1. This example is illustrated in Fig 5.

First, in the upper-left-hand panel of Fig 5, we draw lines to represent the best-fit lines within each case. The slope of each line is equivalent to the coefficient from running a bivariate regression within each time point or case. In this specific example, the lines are parallel because we set each slope to -1. Next, in the upper-middle-panel, we draw lines to represent the best-fit lines within each time point. Again, in this specific example, the lines are parallel because we set each slope to 1. We plot points exactly at the intersections in this lattice in the upper-right-hand panel because TSCS data have the specific restriction that every observation exists in exactly one case and in exactly one time point. The only way to generate data with this property is to draw points at the intersections. Finding these intersections is equivalent to solving the system of equations in 16. Finally, in the bottom-left-hand panel, we add exogenous noise to the outcome of each datapoint—because the errors are uncorrelated with X, no linear model run on these data involves an endogeneity bias. Then, as shown in the bottom-middle-panel, we have constructed a simulated TSCS dataset.

Using this framework we can set the intercepts and slopes to any value so long as the within-case and within-time slopes are not equal (otherwise two lines would have infinitely many intersection points). In addition, the lines for each case and for each time point need not be parallel. In section 1, we allow the within-case and within-time slopes to vary; we generate each set of slopes from a normal distribution with a variance of 0.25. In section 1, we demonstrate that the two-way FE estimator is unidentified when both sets of slopes are parallel: that is, when the within-case slopes do not vary across cases and the within-time slopes do not vary over time.

### Two-way FE coefficients average the within-case and within-time slopes in the data

We repeatedly generate new TSCS data from Eqs 17 and 19. In each iteration, we set the number of cases *N* and the number of time points *T* each to be 30. We generate the case-specific intercepts *α*_*i*_, the time-specific intercepts *α*_*t*_, and the exogenous errors *ε*_*it*_ from standard normal distributions. We generate varying within-case slopes *γ*_*i*_ in Eq 15 from a normal distribution with a mean of -3 and a variance of 0.25. We also draw the within-time slopes, *β*_*t*_ in Eq 14, from normal distributions with variances of 0.25.

**Fig 6:**
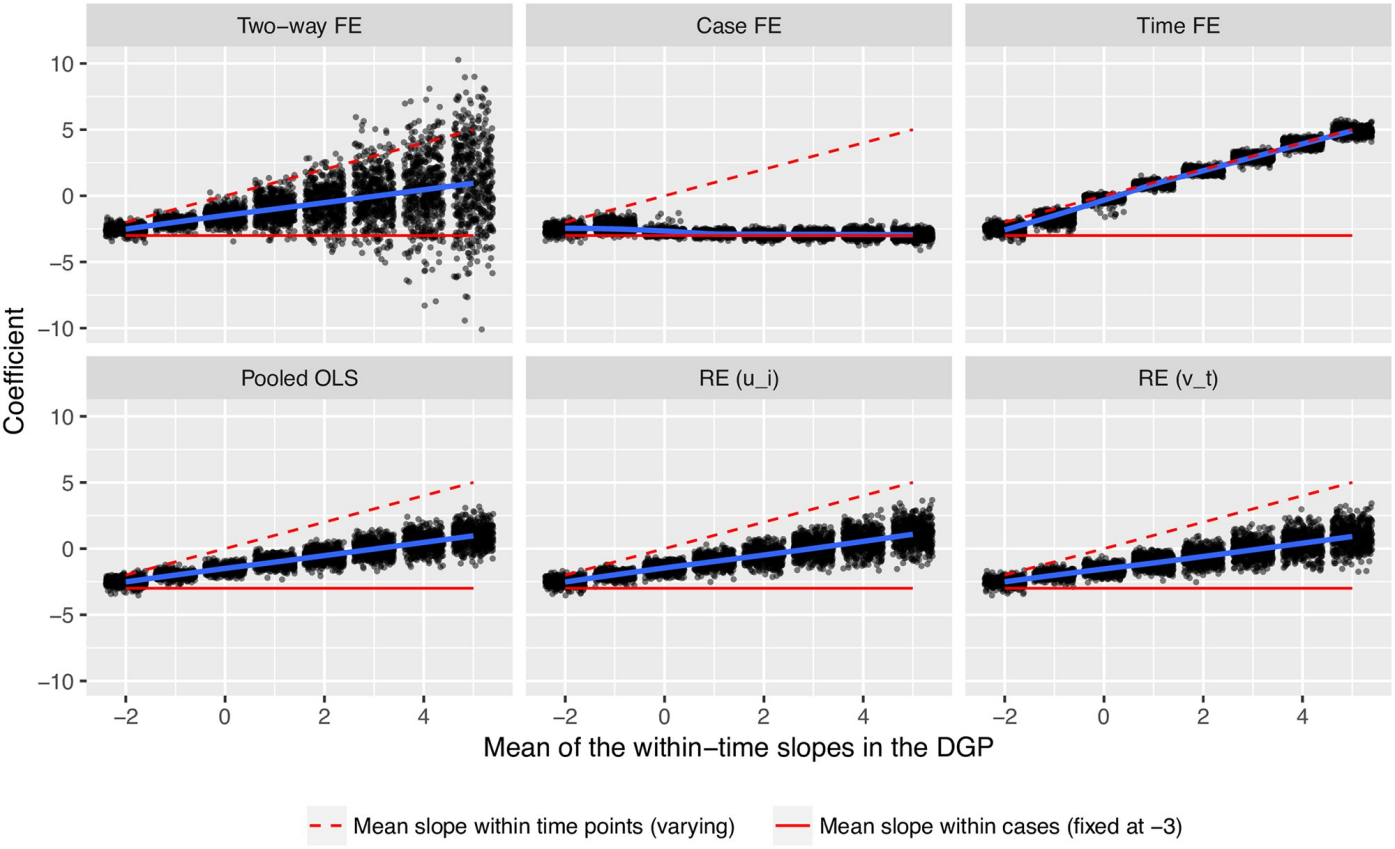
Simulation results, varying the mean of the within-time slopes. For clarity, a small amount of random noise is added to the x-coordinate of each point.

As the experimental treatment, we iteratively set the mean of the within-time slopes to be -2, -1, 0, 1, 2, 3, 4, and 5, and we repeat each simulation 500 times for each of these conditions. We plot these mean values of *β*_*t*_ on the *x*-axis in Fig 6, and we plot the coefficient estimates from each model under consideration on the *y*-axis. We compare the coefficients returned by two-way, case, and time FEs, pooled OLS, and random effects (RE). We run two versions of RE: one that integrates over a case-fixed intercept *u*_*i*_, and one that integrates over a time-fixed intercept *v*_*t*_. The results for each model are aligned in a 3 × 2 grid in Fig 6.

As expected, the case FE coefficient is always about -3, equal to the average within-case slope, despite the fact that the mean of the within-time slopes changes. The time FE coefficients fall along the 45-degree line, indicating that this estimator returns the average within-time slope on average. In contrast, the two-way FE, pooled OLS, and random effects coefficients tend to be estimated in the intermediate space between the mean within-case and mean within-time slopes. The pooled OLS estimator is more efficient in this simulation, but we would note that this does not necessarily demonstrate that the pooled OLS and and random effects estimators are superior to two-way FEs in general as this simulation does not cover the full range of possible kinds of TSCS data, including unbalanced panels that are known to affect pooled OLS and RE. Our intention rather is to show how the two-way FE model is substantively similar to pooled OLS and random effects in that it pools variation across both dimensions.

Fig 6 shows that case FEs are successful in removing the cross-sectional variation so that results clearly describe relationships between variables over time because changes in the within-time slopes do not affect the case FE coefficients. In addition, because time FEs eliminate the temporal variation and model the cross-sectional variation, the time FE coefficients accurately estimate this changing within-time slope regardless of its mean value. In contrast, two-way FE appears to be a pooling estimator like pooled OLS or RE, and depends on both variances.

This result should lead us to reconsider the idea popular in applied work that two-way FEs account for both cross-sectional and temporal variation in the same way that one-way FE models do. As we have shown, two-way FE models are fundamentally different than their one-way cousins despite similarities in the estimating equations. For example, although two-way FEs include a dummy variable for every case, the two-way FE coefficients change along with the within-time slope, as do the coefficients from pooled OLS and both random effects models. Thus, by including time dummies in addition to case dummies, two-way FEs differ substantially from one-way case FEs because this model is once again dependent on both the cross-sectional and temporal variation.

### (Un-)Identifiability of two-way FE estimates

When we use a one-way FE model, we estimate a set of lines—one line for each case or for each time point—with the same slope but with different intercepts, so that these lines are parallel. This means that, unless an interaction is used or the coefficient is explicitly modeled as random, the case FE model assumes that the within-case slopes are fixed across cases and the time FE model assumes that the within-time slopes are fixed across time points.

Whether or not these two assumptions hold, the case FE model and the time FE model are identified and return coefficients as they normally do. In contrast, the two-way FE model is unidentified when the within-case slopes are fixed across cases at the same time as the within-time slopes are fixed across time points. The proof that the two-way FE coefficient is unidentified in this case is as follows:

**Proof.** If the within-time slopes are fixed across time points, then *β*_*t*_ = *β*, ∀*t* in Eq 14, and if the within-case slopes are fixed across cases, then *γ*_*i*_ = *γ*, ∀*i* in Eq 15. Then the system of equations in 16 becomes

$$\begin{array}{c} \left\{ \begin{array}{c} E\left(y_{it}\right)=\alpha_{t}+\beta x_{it},\\ E\left(y_{it}\right)=\alpha_{i}+\gamma x_{it}, \end{array}\right. \end{array}$$ (20)

and the solution to this system is

$$\begin{array}{ccc} x_{it}=\frac{\alpha_{i}-\alpha_{t}}{\beta -\gamma }, & & E\left(y_{it}\right)=\frac{\beta \alpha_{i}-\gamma \alpha_{t}}{\beta -\gamma }. \end{array}$$ (21)

Consider the version of the two-way FE estimator in balanced panels that is listed in Eq 9:

$$\begin{array}{c} {\hat{\beta }}_{TW}=\frac{\sum_{i=1}^{N}\sum_{t=1}^{T}\left(y_{it}-{\bar{y}}_{i}-{\bar{y}}_{t}+\bar{y}\right)\left(x_{it}-{\bar{x}}_{i}-{\bar{x}}_{t}+\bar{x}\right)}{\sum_{i=1}^{N}\sum_{t=1}^{T}{\left(x_{it}-{\bar{x}}_{i}-{\bar{x}}_{t}+\bar{x}\right)}^{2}}. \end{array}$$ (22)

Next, consider just the denominator:

$$\begin{array}{cc} & \sum_{i=1}^{N}\sum_{t=1}^{T}{\left(x_{it}-{\bar{x}}_{i}-{\bar{x}}_{t}+\bar{x}\right)}^{2}\\ = & \sum_{i=1}^{N}\sum_{t=1}^{T}{\left(x_{it}-\frac{\sum_{t=1}^{T}x_{it}}{T}-\frac{\sum_{i=1}^{N}x_{it}}{N}+\frac{\sum_{i=1}^{N}\sum_{t=1}^{T}x_{it}}{NT}\right)}^{2}. \end{array}$$ (23)

We substitute *x*_*it*_ with the solution for *x*_*it*_ in Eq 21,

$$\begin{array}{cc} & \sum_{i=1}^{N}\sum_{t=1}^{T}{\left(\frac{\alpha_{i}-\alpha_{t}}{\beta -\gamma }-\frac{\sum_{t=1}^{T}\frac{\alpha_{i}-\alpha_{t}}{\beta -\gamma }}{T}-\frac{\sum_{i=1}^{N}\frac{\alpha_{i}-\alpha_{t}}{\beta -\gamma }}{N}+\frac{\sum_{i=1}^{N}\sum_{t=1}^{T}\frac{\alpha_{i}-\alpha_{t}}{\beta -\gamma }}{NT}\right)}^{2}\\ = & \sum_{i=1}^{N}\sum_{t=1}^{T}{\left(\frac{\alpha_{i}-\alpha_{t}}{\beta -\gamma }-\frac{\sum_{t=1}^{T}\alpha_{i}-\alpha_{t}}{T(\beta -\gamma )}-\frac{\sum_{i=1}^{N}\alpha_{i}-\alpha_{t}}{N(\beta -\gamma )}+\frac{\sum_{i=1}^{N}\sum_{t=1}^{T}\alpha_{i}-\alpha_{t}}{NT(\beta -\gamma )}\right)}^{2}\\ = & \frac{\sum_{i=1}^{N}\sum_{t=1}^{T}{\left(NT\left(\alpha_{i}-\alpha_{t}\right)-N\sum_{t=1}^{T}\left(\alpha_{i}-\alpha_{t}\right)-T\sum_{i=1}^{N}\left(\alpha_{i}-\alpha_{t}\right)+\sum_{i=1}^{N}\sum_{t=1}^{T}\left(\alpha_{i}-\alpha_{t}\right)\right)}^{2}}{N^{2}T^{2}{\left(\beta -\gamma \right)}^{2}}\\ = & \frac{\sum_{i=1}^{N}\sum_{t=1}^{T}(NT\alpha_{i}-NT\alpha_{t}-N\sum_{t=1}^{T}\alpha_{i}+N\sum_{t=1}^{T}\alpha_{t}-T\sum_{i=1}^{N}\alpha_{i}+T\sum_{i=1}^{N}\alpha_{t}+\sum_{i=1}^{N}\sum_{t=1}^{T}\alpha_{i}-\sum_{i=1}^{N}\sum_{t=1}^{T}\alpha_{t}{)}^{2}}{N^{2}T^{2}{\left(\beta -\gamma \right)}^{2}}\\ = & \frac{\sum_{i=1}^{N}\sum_{t=1}^{T}{\left(NT\alpha_{i}-NT\alpha_{t}-NT\alpha_{i}+NT{\bar{\alpha }}_{t}-NT{\bar{\alpha }}_{i}+NT\alpha_{t}+NT{\bar{\alpha }}_{i}-NT{\bar{\alpha }}_{t}\right)}^{2}}{N^{2}T^{2}{\left(\beta -\gamma \right)}^{2}}\\ = & \frac{\sum_{i=1}^{N}\sum_{t=1}^{T}(\left[NT\alpha_{i}-NT\alpha_{i}\right]+\left[NT\alpha_{t}-NT\alpha_{t}\right]+\left[NT{\bar{\alpha }}_{t}-NT{\bar{\alpha }}_{t}\right]+\left[NT{\bar{\alpha }}_{i}-NT{\bar{\alpha }}_{i}\right]{)}^{2}}{N^{2}T^{2}{\left(\beta -\gamma \right)}^{2}}=0. \end{array}$$ (24)

Since the denominator of the two-way FE estimator must be 0 under these conditions, it follows that the two-way FE model is unidentified.

This un-identifiability will manifest itself as a non-full-rank matrix that will result in an error in statistical software packages when matrix inversion is attempted. This problem will occur even if substantial variation exists in both the over-time and cross-section dimensions of the dataset; in other words, this problem is not a degrees of freedom issue.

The reason that this un-identifiability has not been recognized before is because Stata and R can deal with the fact that this model is unidentified by automatically selecting one of the fixed effects to drop from the model, as can also occur with the more common issues of multicollinearity and missing data. As such, this particular problem has likely been ignored when it occurs as it is difficult to impossible to diagnose without running regressions within each time point or case. Generally speaking, if the dropped FE happens to be a dummy variable for a case, then the resulting coefficient on *x* resembles the time FE coefficient on *x*. If the dropped FE happens to be a dummy variable for a time point, the coefficient resembles the case FE coefficient on *x*. In both Stata and R, we have noticed that the FE to drop in the case of non-identification is determined in part by the order in which the FEs are entered into the formula to run the linear model, although this of course depends on which estimation command is used. It is important for researchers to pay close attention to whether or not any FEs are dropped in the final model results, as this issue may be due to model non-identification.

Furthermore, this un-identifiability can manifest itself in very unstable estimates of ${\hat{\beta }}_{TW}$ when the within-case slopes are nearly equal across cases and when the within-time slopes are nearly equal across time points. That is, when the variance of the within-case slopes across cases and the variance of the within-time slopes across time points are both close to 0, the variance of ${\hat{\beta }}_{TW}$ approaches infinity. To demonstrate this behavior, we use another simulation.

**Fig 7:**
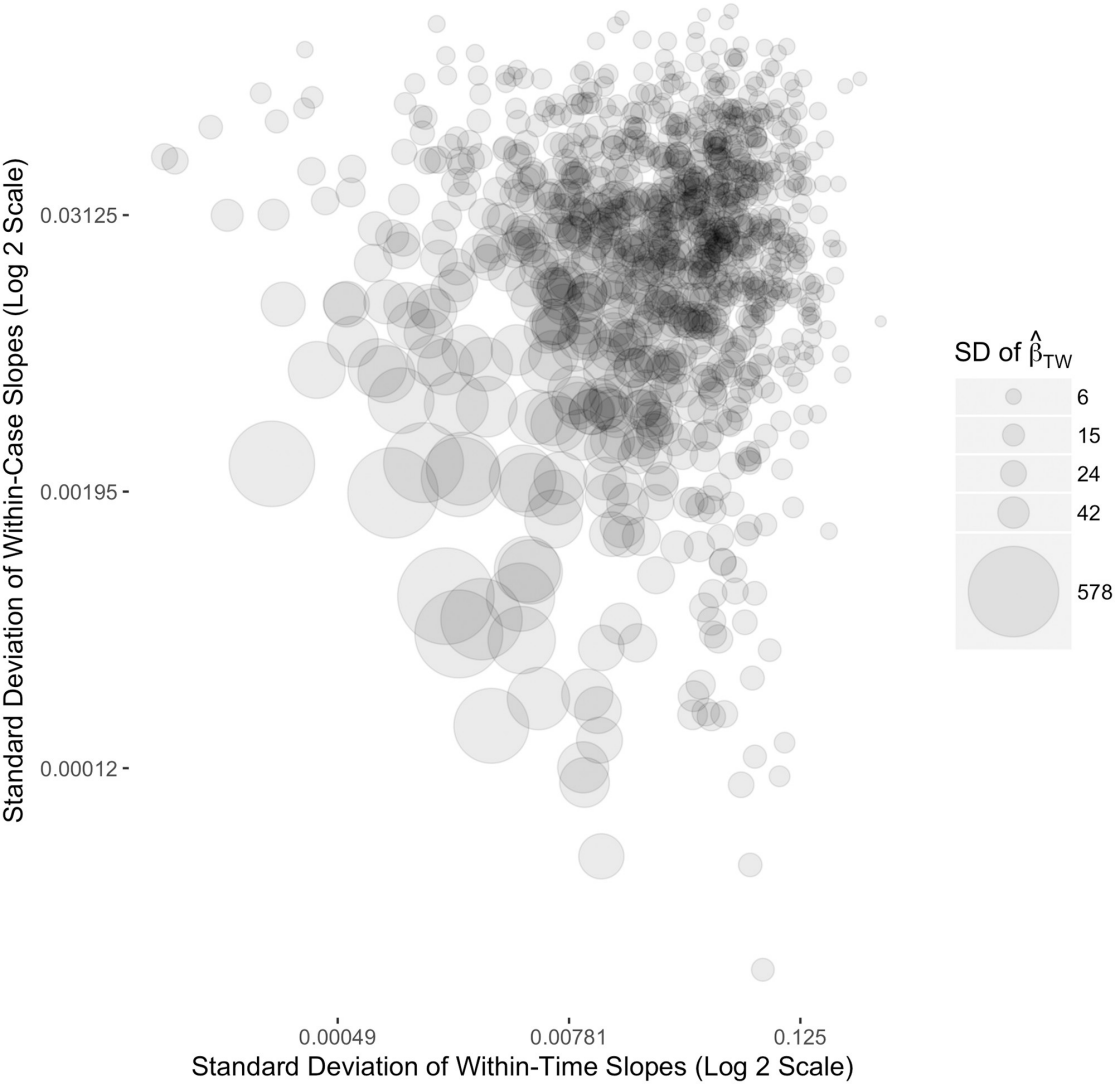
Two-way FE estimates with almost-fixed within-case and within-time slopes. The value of ${\hat{\beta }}_{TW}$ is equal to the coefficient on *x* obtained from a two-way FE linear regression of the simulated data. The standard deviation of the ${\hat{\beta }}_{TW}$ estimates is calculated from 100 random replicates for the given values of the standard deviation of the within-case and within-time slopes along the *x* and *y* axis.

We use the procedure described in section 1 to generate TSCS datasets with 30 cases and 30 time points each, where case-specific and time-specific intercepts and exogenous error are drawn from standard normal distributions. For each dataset, we set the mean of the within-case slopes to be -3 and the mean of the within-time slopes to be 3. In this simulation, we change the standard deviation of the within-case and within-time slopes as an experimental treatment. We draw the standard deviations of the slopes from exponential distributions with rate parameters set at 25 so that the randomly generated standard deviations are clustered at or near zero. For each pair of drawn standard deviations, we generate 100 TSCS datasets, we run the two-way FE model on each dataset, and we record the 100 coefficient estimates. We report the standard deviation of these coefficients, conditional on the standard deviations of the within-case and within-time slopes, in Fig 7. We repeat the process 1000 times.

Fig 7 is a scatterplot in which the *x*-axis represents the standard deviation of the within-time slopes and the *y*-axis represents the standard deviation of the within-case slopes. The size of each dot on this graph represents the standard deviation of the two-way FE coefficients estimated across the 100 TSCS datasets with the values of the standard deviations for the within-case and within-time slopes along the axes. As can be seen, as the standard deviation of the within-case and within-time slopes approaches zero, the standard deviation of the coefficients ${\hat{\beta }}_{TW}$ from the two-way FE model converges to infinity. With truly fixed slopes, the model matrix is singular and it is not possible to return a coefficient.

What this simulation reveals is that the variance of two-way FE coefficients will increase dramatically as slopes are nearly fixed across cases and time points, and this could well result in very unstable estimates for a particular dataset. The implication of this result is that if we begin with the assumptions that many researchers bring to the two-way FE model—that it represents a single estimate of X on Y, with no heterogeneity in the effect, while accounting for unit-level heterogeneity and time shocks—the two-way FE specification is in fact statistically unidentified. For these reasons, in addition to the issues we have brought up in this paper, we would urge applied researchers to be very careful when employing this model for any TSCS dataset without strong prior knowledge about effect heterogeneity in the cross-section or over-time dimensions of variance.

We would emphasize that this unidentifiability is *not* due to the well-known issue with fixed effects and limited variability in *y*_*it*_ along either cases or time points. Rather, the lack of variability is in the “true” effects of *x*_*it*_, which is what makes the unidentifiability so difficult if not impossible to diagnose without running a regression with multi-collinearity checks turned off.

In S2 Appendix we analyze the empirical question of the relationship between democracy and economic development, which has received much attention in the panel data literature. We refer the interested reader there to see the practical ramifications of re-interpreting fixed effects models with respect to a real research question where one-way and two-way fixed effects models have widely varying results.

## Discussion: Choosing the right model for the research question

Fixed effects models address omitted variable bias by accounting for time-fixed covariates, case-fixed covariates, or both. But they also change the research question being evaluated by the model. It is important for researchers to be aware of both of these implications of a FE specification, to pay attention to how coefficients from the model should be interpreted, and to choose a model that provides an answer to the desired research question.

We therefore issue a recommendation to applied researchers. We suggest that research questions be phrased in a way that makes the principal comparison being made explicitly clear. We then urge researchers to choose a model whose interpretation matches the intended research question. In particular, we encourage researchers to phrase research questions in a way that is more specific than “what is the effect of *x* on *y*?” In TSCS data, any answer to this general question pools across a comparison of cases and a comparison of time points, and we have to assume that the way that cases compare to one another—whether they are countries, U.S. states, political parties, institutions, elites, or individual survey respondents—is equal to the way that they compare to themselves over time. Such an assumption does not respect the nuanced theories that social scientists develop about these cases or about how they change over time.

The interpretation of a time FE model corresponds to a research question that involves cross-sectional comparisons, and the interpretation of a case FE model corresponds to a research question that involves comparisons over time. We emphasize, however, that this heuristic does not solve the estimation problems that can be manifest in TSCS data. The time FE estimator places the analysis in a cross-sectional context, and all of the problems of cross-sectional work may be present. The case FE estimator places the analysis in a time series context, and statistical issues with time series data must be addressed. Given that some TSCS methods add significant complexity to models, we believe it is best to start with a solid basis of what the effect of *x* is in a well-defined estimation so that the changes produced by any model extensions can be understood with reference to a simpler base model. Once we start with the goal of estimating an effect in the cross-sectional or time dimension rather than forcing the two to be somehow combined to produce one estimate, then it is easier to see what problems still exist that might obscure the relationship under study.

This approach also provides a useful framework for elaborating upon the one-way FE models to describe more complex and interactive comparisons. For example, interactive fixed effects models have become a useful way of exploring conditional relationships in panel data [22], and our analysis helps elucidate these more sophisticated approaches as well. Furthermore, it encourages the development of new TSCS methods since a method may have a useful application without having to be the best, catch-all approach for estimation on both dimensions and any combination of the dimensions.

## Conclusion

The two-way fixed effects model, an increasingly popular method for modeling TSCS data, is substantively difficult to interpret because the model’s estimates are a complex amalgamation of variation in the over-time and cross-sectional effects. While one-way FE models can be understood as generalizations of the effects that exist within one case or within one time point, the two-way FE model can only be understood as a generalization of the effect of deviations from the case-means at a particular point in time, or equivalently, as a generalization of the effect of deviations from the time-means for each particular case. This interpretation of two-way FE coefficients is accurate, but is usually difficult to conceptualize and to communicate, and seldom matches the questions researchers intend to answer.

Because of the restrictive assumptions and difficulty in substantive interpretation, we do not recommend that applied researchers rely on the two-way FE model except for situations in which the assumptions are well-understood, such as the canonical difference-in-difference design.

We hope that an increased emphasis on interpretation leads methodologists and substantive researchers to think about TSCS data in a new way. Instead of beginning with the standard linear model and applying a myriad of corrections to account for the features of TSCS data, we suggest an approach that builds up to a complete and meaningful model from simple constituent parts. A researcher must first define the essential comparison in the data: the difference between two cases at a particular point in time, the difference between two points in time for a particular case, or a more complex and interactive comparison if the question calls for one and if such a comparison can be clearly described. The researcher must then choose how to pool across all of these comparisons to generalize a finding and employ the power in the data. Future work to develop TSCS methods will be most useful to applied researchers if the method is clear about what it compares and how it generalizes across comparisons.

## Supporting information

S1 Appendix How the two-way FE estimator compares to a difference-in-difference design. We further explicate on the differences-in-differences model showing algebraically the relationship between the two specifications.
(PDF)

S2 Appendix Empirical illustration: Economic development and democracy. In this appendix we replicate and extend recent findings by Acemoglu et al. and Haber and Menaldo on the relationship between economic development (GDP and oil resources) and democracy, showing how the application of 2-way FE models leads to incorrect conclusions about null effects of GDP/oil on democracy.
(PDF)

S3 Appendix Additional proofs. In this appendix we offer additional analysis of 1-way FE estimators as weighted averages and a derivation of the two-way FE estimator in balanced panels.
(PDF)
