# Supplementary material for: Interpretation and identification of within-unit and cross-sectional variation in panel data models
Source: PLoS One. 2020 Apr 21;15(4):e0231349. doi: 10.1371/journal.pone.0231349 (PMC7173782; doi:10.1371/journal.pone.0231349)
Supplement: S1 Appendix — We further explicate on the differences-in-differences model showing algebraically the relationship between the two specifications. (PDF) [file pone.0231349.s001.pdf]

S1 Appendix: How the Two-Way FE Estimator Compares to a Difference-in-Difference Design

Jonathan Kropko
School of Data Science
University of Virginia
jkropko@virginia.edu

Robert Kubinec
Department of the Social Sciences
New York University Abu Dhabi
rmk7@nyu.edu
(corresponding author)

March 8, 2020

As we discussed in the main article, the two-way FE model is often defended as a difference-in-difference (DiD) design. However, in order to be an estimator for a DiD effect, the two-way FE model must make assumptions that are more unrealistic than researchers who are interested in causal inference would typically be willing to accept when the data contain more than two time points, the treatment is not binary, or when the treatment is not zero for all cases in the first time point.

A DiD design approximates random assignment in a time period by subtracting from the outcome the value of the outcome at a prior time point (Morgan and Winship 2007, 253-254). The canonical application of a DiD estimator is to data that contain two time points, $t \in \{1, 2\}$, and a binary treatment variable X_{it} that is 0 for all cases in time point 1, 0 for the control group in time point 2, and 1 for the treatment group in time point 2. If we denote the outcomes for cases in the control group to be y_{1t} and the outcomes for the cases in the treatment group to be y_{2t}, then the DiD estimate is given by

$$\delta = E(y_{22}) - E(y_{12}) - E(y_{21}) + E(y_{11}). \tag{1}$$

Consider the two-way FE regression from equation **??**,

$$y_{it} = \alpha_i + \alpha_t + \beta x_{it} + \varepsilon_{it}.$$

Under the same conditions as the canonical DiD application, this regression yields a coefficient estimate that is equal to δ. Again, assume there are two time periods, a control group represented by $i = 1$ and a treatment group represented by $i = 2$, and a treatment given by

$$x_{it} = \begin{cases} 1 & \text{if } i = 2 \text{ and } t = 2, \\ 0 & \text{otherwise.} \end{cases} \tag{2}$$

Then the DiD statistic is

$$\begin{aligned}\delta &= E(y_{22}) - E(y_{12}) - E(y_{21}) + E(y_{11}) \\ &= (\alpha_{i=2} + \alpha_{t=2} + \beta) - (\alpha_{i=1} + \alpha_{t=2}) - (\alpha_{i=2} + \alpha_{t=1}) + (\alpha_{i=1} + \alpha_{t=1}) \\ &= \beta.\end{aligned}$$

Therefore, under these ideal conditions, the two-way FE estimator is a DiD estimator.

The two-way FE estimator is often proposed as a DiD design even when there are multiple time points and when the treatment is continuous rather than binary. Our goal is to characterize how exactly a DiD estimate must be defined in this general context in order for β from a two-way FE model to remain equal to δ. Without loss of generality, let there be any two distinct time points s and t that are not necessarily just one unit of time apart, and let there be two distinct cases i and j. If $(x_{it} - x_{js}) - (x_{is} - x_{js}) = d$, then the generalized DiD estimate is

$$\begin{aligned}\delta &= E(y_{it}) - E(y_{jt}) - E(y_{is}) + E(y_{js}) \\ &= (\alpha_i + \alpha_t + \beta x_{it}) - (\alpha_j + \alpha_t + \beta x_{jt}) - (\alpha_i + \alpha_s + \beta x_{is}) + (\alpha_j + \alpha_s + \beta x_{js}) \\ &= \beta(x_{it} - x_{js} - x_{it} + x_{js}) \\ &= d\beta.\end{aligned}$$

In particular, if $d = 1$ then $\beta = \delta$.[1] Therefore the two-way FE coefficient also has the following substantive interpretation:

> Consider two distinct cases i and j and two distinct time points s and t, and let $d = x_{it} - x_{is} - x_{jt} + x_{js}$. A one-unit increase in d is associated with a β change in the DiD statistic $\delta = E(y_{it}) - E(y_{is}) - E(y_{jt}) + E(y_{js})$, on average.

Let's consider the tacit assumptions one makes when using this coefficient to characterize an effect. In addition to the usual difference-in-difference assumption of parallel paths (see, for example, Baltagi 2011), this interpretation requires an assumption of homogeneity across both cases and time points. Homogeneity across cases is often non-controversial, especially if there is no reason to expect an interaction to exist in the cross-section; it is the assumption we make any time we allow an effect to generalize across cases. But this DiD effect also assumes homogeneity across time in a way that does not model temporal processes. The two time points s and t may exist at any point in the time series and they do not have to be adjacent. In other words, this effect generalizes across all time differences, regardless of whether they occur early in the time series, later in the time series, whether they are one year apart, or 100 years apart. Because the effect we estimate generalizes across cases and time points, we must rely heavily on the linearity of the model itself to impute the comparisons that we do not observe directly. Therefore an analyst must be convinced that the true causal estimate is both homogeneous across time and linear in order for the two-way FE coefficient to be an accurate representation of the DiD statistic.

We argue that these assumptions are more restrictive than researchers who attempt to achieve causal identification would be willing to accept. Causal inference methodology is founded on the principle that

1. Note that this property of δ also holds when the true model is one-way case FEs, one-way time FEs, or pooled OLS, as these models are special cases of the two-way FE model where one or both sets of FEs are zero.

before any estimation can occur, a researcher must clearly describe the causal effect of interest. In the case of a continuous treatment, the researcher must be clear about how different regions on the continuum impact the outcome differently, or else defend the linearity assumption. In the case of time dependent data, this specification must be clear about when an effect occurs and how long it takes to occur. Morgan and Winship (2007, 274) make this point explicit with regard to TSCS data:

> Defendable assumptions about the treatment assignment process must be specified. And, to use longitudinal data to its maximum potential, researchers must carefully consider the dynamic process that generates the outcome, clearly define the causal effect of interest, and then use constrained models only when there is reason to believe that they fit the underlying data.

Difference-in-difference estimation is a causal inference strategy, but the practice of employing a two-way FE model as a difference-in-difference design does not responsibly address the concerns regarding the model's assumptions. Other causal inference strategies, such as matching, dispel the assumption of linearity that the two-way FE estimator strongly makes. Approaches such as the synthetic control method (Abadie and Gardeazabal 2003; Xu 2017) are explicit with regard to the comparisons they make, both cross-sectionally and over time. We therefore refer researchers to methods like these that are more careful about the assumptions they employ.

References

Abadie, Alberto, and Javier Gardeazabal. 2003. "The Economic Costs of Conflict: A Case Study of the Basque Region." *The American Economic Review* 93 (1): 113–132.

Baltagi, Badi H. 2011. *Econometrics.* Fifth. Springer.

Morgan, Stephen L., and Christopher Winship. 2007. *Counterfactuals and Causal Inference: Methods and Principles for Social Research.* New York: Cambridge University Press.

Xu, Yiqing. 2017. "Generalized Synthetic Control Method: Causal Inference with Interactive Fixed Effects Models." *Political Analysis* 25 (1): 57–76.
