# Supplementary material for: Interpretation and identification of within-unit and cross-sectional variation in panel data models
Source: PLoS One. 2020 Apr 21;15(4):e0231349. doi: 10.1371/journal.pone.0231349 (PMC7173782; doi:10.1371/journal.pone.0231349)
Supplement: S2 Appendix — In this appendix we replicate and extend recent findings by Acemoglu et al. and Haber and Menaldo on the relationship between economic development (GDP and oil resources) and democracy, showing how the application of 2-way FE models leads to incorrect conclusions about null effects of GDP/oil on democracy. (PDF) [file pone.0231349.s002.pdf]

S2 Appendix Empirical Illustration: Economic Development and Democracy

Jonathan Kropko
School of Data Science
University of Virginia
jkropko@virginia.edu

Robert Kubinec
Department of the Social Sciences
New York University Abu Dhabi
rmk7@nyu.edu
(corresponding author)

March 8, 2020

1 Introduction

In this appendix we examine an existing research question to provide a case study for how to use and apply FE models most fruitfully. We look at one of the oldest questions in social science: is economic development a necessary precursor to democratization? While this hypothesis can originally be found in Marxist thinking about the necessity of the growth of capitalism before the advent of democracy (Moore 1966), more recent scholarship has tested this proposition using ever larger datasets of country-year level measures of democracy and economic growth.

Given that these data naturally have both over-time variation and between-country (cross-sectional) variation, scholars have examined this question using a variety of statistical models, including linear models with case FEs (Kennedy 2010) and two-way FEs (Acemoğlu et al. 2008; Boix 2011; Haber and Menaldo 2011). But there is as yet no general consensus on the relationship between GDP growth and democratization, with some positing an intermediating effect of inequality (Acemoğlu and Robinson 2006; Houle 2009; Boix and Stokes 2003), others the intermediating effect of fuel exports (Haber and Menaldo 2011; Andersen and Ross 2014), and still others that the relationship is unconditionally positive, with economic development inevitably leading to democratization (Inglehart and Welzel 2005). Finally, there are skeptics who argue that there is in fact no effect of GDP growth on democratization, but rather on democratic consolidation (Limongi and Przeworski 1997).[1] Because different researchers have employed different methods on the same data, debates in this literature are closely linked to debates over methods. Fundamentally, applied researchers do not agree on whether the data support the conclusion that these variables are related to each other, and if so, in what direction.

Our objective is not to resolve all questions in this broad research literature, but rather to show that our ability to learn about the research question from the available data depends on our understanding of how different models speak to the different dimensions of variation in the data. One-way FE models have the advantage of providing clear interpretations that unambiguously speak to the cross-sectional or

1. For a full review of this literature, see Coppedge (2012) and Ross (2015).

temporal variances, but not both. In contrast, two-way FE models are much more difficult to interpret in an applied setting, which means that we are unable to answer the research question even with suitable data.

In this appendix, we first replicate two well-known panel data papers and then re-estimate specifications with new data from the Varieties of Democracy project (V-DEM).

2 Replication of Acemoğlu et al. (2008) and Haber and Menaldo (2011)

To apply the one-way and two-way FE models to the examination of the effect of economic development on democracy, we focus on two well-known and heavily cited studies in this literature: Acemoğlu et al. (2008) and Haber and Menaldo (2011). Acemoğlu et al. (2008) employ a two-way FE model to assess the effect of GDP on democracy, and Haber and Menaldo (2011) employ a two-way FE model to assess the effect of fuel resources (a subset of GDP) on democracy. Both report counter-intuitive findings: Acemoğlu et al. (2008) conclude that GDP has no relationship to democratization, while Haber and Menaldo (2011) suggest that increased oil revenues have a positive, not negative, effect on democratization. Although both articles phrase their research questions in terms of the over-time effect, both use two-way FEs and justify this approach as a method to eliminate trends and unobserved omitted variables. These counter-intuitive findings, however, are dependent on the use of two-way FEs and do not manifest when one-way FEs are used instead. Therefore the choice of FE specification can lead to drastically different substantive conclusions.

Both of these papers are well-known (collectively cited 1,640 times in Google Scholar), both use two-way FE models, and both have produced results at odds with common wisdom. Acemoğlu et al. (2008) have argued that GDP has no relationship to democratization, while Haber and Menaldo (2011) have shown that increased oil revenues have a positive, not negative, effect on democratization. We replicate their work and reanalyze their data using one-way FEs in addition to two-way FEs.

Although Haber and Menaldo are interested in "variance within countries over time" (p. 2), which indicates an interest in the over-time effect, they include year FEs in addition to country (case) FEs in all of their models (p. 15). Similarly, Acemoğlu et al. have the same interest in the over-time effect and use year FEs to control for possible omitted variables. Because the models include a dummy variable for each country and each time point, these models are two-way FE models. We replicated the main findings of both papers in Tables 1 and 2. We compare and contrast their two-way FE models with one-way FE models for either countries or years.

Table 1: Replication and Re-Analysis of Acemoğlu et al. (2008) Table 2

	Pooled OLS	Two-way FE	One-way Year FE	One-way Country FE
Democracy$_{t-1}$	0.692	0.379	0.706	0.394
	(0.63, 0.76)	*(0.28, 0.48)*	*(0.64, 0.78)*	*(0.30, 0.49)*
**Log GDP Per Capita**$_{t-1}$	**0.080**	**0.010**	**0.072**	**0.074**
	(0.06, 0.10)	*(-0.06, 0.08)*	*(0.05, 0.09)*	*(0.03, 0.12)*
R-Squared	0.71	0.80	0.73	0.78
Observations	945	945	945	945

Table 2: Replication and Re-analysis of Haber and Menaldo (2011) Table 5

	Two-way FE	One-way Country FE	One-way Time FE
Polity in Levels$_{t-1}$	-0.087	-0.085	-0.046
	(-0.102, -0.072)	*(-0.100, -0.070)*	*(-0.055, -0.037)*
**Total Oil Income$_{t-1}$**	**0.055**	**0.027**	**-0.074**
	(0.017, 0.093)	*(-0.009, 0.062)*	*(-0.128, -0.020)*
△ **Total Oil Income**	**-0.020**	**-0.002**	**-0.089**
	(-0.062, 0.022)	*(-0.037, 0.033)*	*(-0.155, -0.024)*
Log(Per Capita Income)$_{t-1}$	-0.279	0.104	0.206
	(-0.923, 0.364)	*(-0.380, 0.588)*	*(-0.101, 0.513)*
Civil War$_{t-1}$	0.065	0.304	-0.084
	(-0.838, 0.968)	*(-0.614, 1.223)*	*(-0.942, 0.773)*
Regional Democratic Diffusion$_{t-1}$	0.025	0.021	0.025
	(0.011, 0.040)	*(0.008, 0.033)*	*(0.017, 0.034)*
Global Democratic Diffusion$_{t-1}$	0.058	0.100	-0.062
	(-0.001, 0.117)	*(0.057, 0.143)*	*(-0.078, -0.046)*
△ Log(Per Capita Income)	1.289	-0.487	1.631
	(-2.206, 4.785)	*(-3.707, 2.733)*	*(-2.045, 5.307)*
△ Regional Democratic Diffusion	0.375	0.366	0.373
	(0.234, 0.516)	*(0.228, 0.504)*	*(0.232, 0.515)*
△ Global Democratic Diffusion	-0.277	0.138	-0.124
	(-0.496, -0.058)	*(0.002, 0.273)*	*(-0.329, 0.081)*
Observations	10, 195	10, 195	10, 195
R-squared	0.098	0.078	0.072
Number of groups	163	163	163

It is clear that the two-way estimates are different from the one-way FE models, although the underlying drivers of this difference are not clear. For Haber and Menaldo, the two-way effect appears to converge to the one-way case FE effect. However, for Acemoğlu et al., the two-way effect is close to zero while both one-way models have positive and statistically significant coefficients. Thus these two-way estimates reflect a nonlinear combination of the one-way case and time effects, but with additional variance from the pooled model that will ensure that it is never equal to either the case or time coefficient.

Both papers have DVs that are measured with the popular democracy score indexes Freedom House and Polity IV. These two outcomes do not vary much over time, in large part because they are measured as ordinal categories and we do not observe countries moving from one category to another very often. The histograms for the DVs in both papers are shown in Figure 1. Acemoğlu et al. use Freedom House scores and Haber and Menaldo use a differenced Polity IV score, both of which tend to cluster at certain

values along the scale. For the differenced Polity IV scale, zero is the dominant value in the dataset, representing an observation in which the score did not change from year to year.

Figure 1: Histogram of Freedom House Scores and Differenced Polity Scores

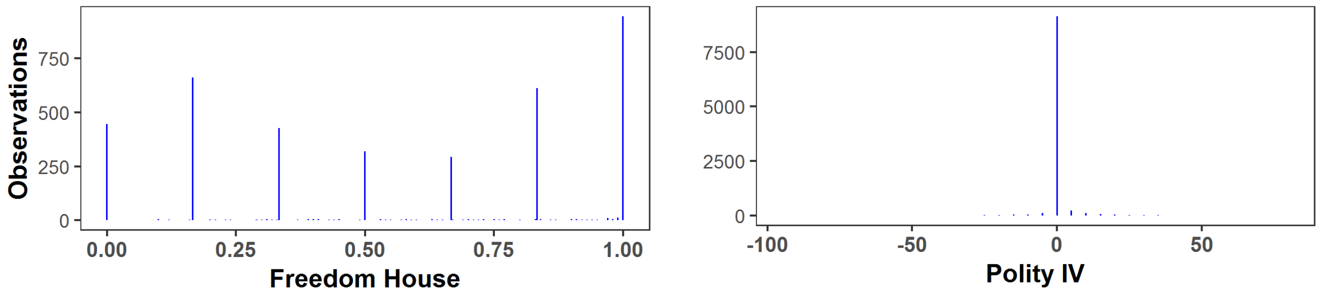

This lack of temporal variation in the DV is problematic for case FEs, which only work with the time series. But in a given year there is plenty of variation in regime type across countries, so time FEs do not suffer from the same issues as case FEs since this approach only works with the cross-sections. Because the two-way FE model incorporates both dimensions of variance, the lack of temporal variation in the DV may effect two-way FE coefficients in unpredictable ways, such as deflating to zero in the Acemoğlu et al. data.

3 Re-analysis with V-DEM Data

In this section we re-analyze their findings using more recent data.

To do so, we employ data from the Varieties of Democratization Project's (V-DEM) Electoral Democracy Index (Coppedge et al. 2017). This new index advances the field of democratization research by utilizing multiple indicators of electoral democracy in an item-response model to produce a democracy index that takes into account measurement uncertainty. We use both independent variables of interest from Acemoğlu et al. (2008) and Haber and Menaldo (2011): GDP, and fuel resources, which includes oil and natural gas. The results for the country FE, year FE and two-way FE models for the regression of GDP and fuel resources on democracy are shown in Table 3.[2]

These results show how case and time FEs can differ from a two-way FE model. The effect of GDP on democracy is positive and very similar for both one-way models: for every log increase in GDP, the V-DEM democracy index rises about 0.2 points. However, for fuel resources, the time effect is nearly five times larger than the case effect. In other words, an increase of fuel resources within a country over time has a negative but small effect on democratization, while an increase of fuel resources relative to other countries has a much larger and negative effect on democratization. Even though the same variable is being measured either within countries or time points, the effects will differ because they represent a fundamentally different dimension of variation.

By contrast, what is notable from Table 3 is the small and, in the case of fuel resources, statistically insignificant estimates of the coefficients of GDP and GDP-oil for the two-way FE models. This pattern

2. We followed Bizarro, Coppedge, and Pemstein (2016)'s advice for incorporating measurement uncertainty by bootstrapping the regression coefficients over the dependent variable's MCMC iterations. Clustered standard errors by country are computed using the R *multiwayvcov* package.

Table 3: GDP, Oil and Democracy Model Results

Model	Log GDP	SE Log GDP	Fuel Income, Thousands	SE Fuel Income
GDP Case Effects	0.191	0.013		
	(0.165, 0.216)			
GDP Time Effects	0.166	0.015		
	(0.136, 0.196)			
GDP Two-Way Effects	0.060	0.023		
	(0.014, 0.106)			
GDP-Oil Case Effects	0.184	0.014	-0.544	0.249
	(0.157, 0.211)		*(-1.020,-0.046)*	
GDP-Oil Time Effects	0.183	0.013	-2.624	0.355
	(0.157, 0.208)		*(-3.320,-1.929)*	
GDP-Oil Two-way Effects	0.079	0.023	-0.126	0.190
	(0.034, 0.125)		*(-0.497, 0.246)*	

Bootstrapped V-DEM 95% confidence intervals and standard errors, which include uncertainty related to measurement error, are included in the table. Standard errors were also clustered by countries.

matches the findings from Acemoğlu et al. (2008) and Haber and Menaldo (2011) that show smaller effects when both sets of FEs are included. It would appear in a two-way FE model that there is no relationship between GDP and democracy, even though the one-way FE models–whether case or time FE models–do show such a relationship. It is clear through this re-analysis how the two-way FE model is *not* a simple average of the one-way FE models, but rather represents an entirely different (and we argue, largely un-interpretable) estimate. In this empirical question, the choice of FE model will radically change one's assessment of what the data are saying. It is a mistake to interpret the coefficients of a two-way FE model as if they speak directly to how cases change over-time.

What can we learn from the preceding analysis? If we search for the *singular* effect of x on y by disregarding the dimension on which the effect exists, then we must conclude that the true effects are small or nonexistent and that the larger one-way FE estimates are artifacts of omitted variable biases. If however we consider how the results inform us about comparisons over time and comparisons drawn between countries, then we must not interpret the two-way FE estimates as more robust versions of one-way FE estimates. The one-way FE estimates are unambiguous averages of the coefficients we derive by looking within specific countries or within specific years. We can learn much more from this analysis if we investigate the dynamics within each country and each year by regressing democracy on GDP using just the data for one country or for just one year.[3] These coefficients are shown in Figures 2a and 2b. The country FE coefficient is drawn as a dotted line in Figure 2a and the year FE coefficient is drawn as a dotted line in Figure 2b. Each dotted line is approximately equal to the average of the individual points on each graph.

3. These one-way FE estimates can be disaggregated either by running independent regressions for each case or year, or by interacting the independent variable of interest with the case or time FEs.

Figure 2: Regression of GDP on Democracy

(a) Coefficients Within Individual Countries

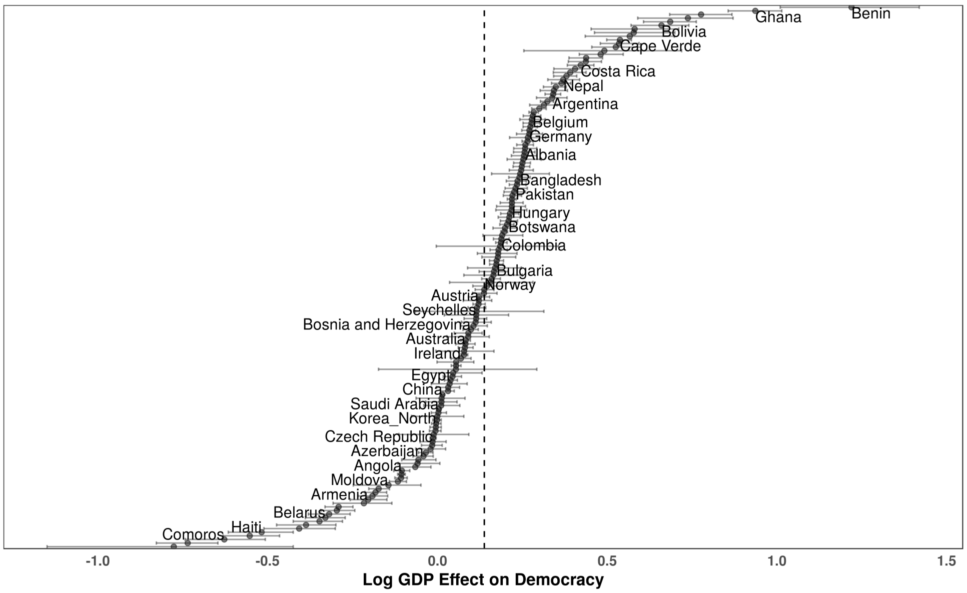

(b) Coefficients Within Individual Years

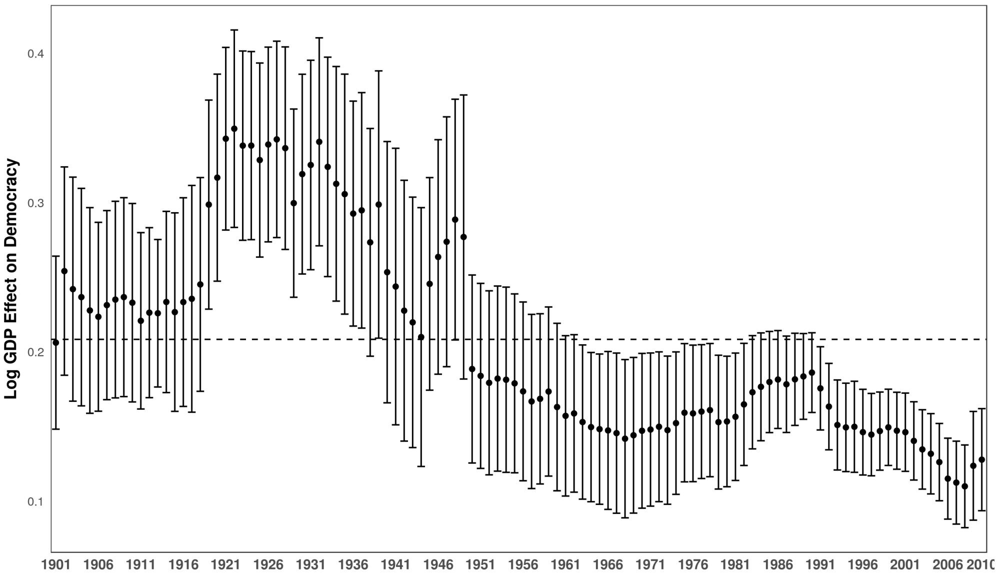

Note: Dotted line indicates the case FE or time FE coefficient from table 3. 95% clustered confidence intervals are included.

Even though the one-way FE coefficients for time points and cases are approximately the same, the disaggregated effects are remarkably different: the relationship between GDP and democracy over time is positive for some countries and negative for others; but GDP is positively related to democracy when comparing countries to one another across the entire time series. Thus even though two different FE models may produce the same coefficient, that does not mean that the underlying effects are equal; the disaggregated estimates may differ even if the averages are superficially equal. These results suggest that more fruitful models should allow over-time effects to vary across countries, or cross-sectional effects to vary over time.

References

Acemoğlu, Daron, Simon Johnson, James A. Robinson, and Pierre Yared. 2008. "Income and Democracy." *American Economic Review* 98 (3): 808–842.

Acemoğlu, Daron, and James Robinson. 2006. *Economic Origins of Dictatorship and Democracy.* Cambridge University Press.

Andersen, Jorgen J., and Michael L. Ross. 2014. "The Big Oil Change: A Closer Look at the Haber-Menaldo Analys." *Comparative Political Studies* 47 (7): 993–1021.

Bizarro, Fernando, Michael Coppedge, and Daniel Pemstein. 2016. *Incorporating V-Dem's Uncertainty Estimates in Regression Analysis.* Working Paper. Visited on August 11, 2016. `https://kellogg.nd.edu/projects/vdem/files/2016/tutorial.pdf`.

Boix, Carles. 2011. "Democracy, Development, and the International System." *American Political Science Review* 105 (4): 809–828.

Boix, Carles, and Susan Carol Stokes. 2003. "Endogenous Democratization." *World politics* 55 (4): 517–549.

Coppedge, Michael. 2012. *Democratization and Research Methods.* Cambridge University Press.

Coppedge, Michael, John Gerring, Staffan I. Lindberg, Svend-Erik Skaaning, Jan Teorell, David Altman, Michael Bernhard, et al. 2017. *V-DEM Dataset v7.* Working Paper. Social Science Research Network. `https://papers.ssrn.com/sol3/papers.cfm?abstract_id=2968289`.

Haber, Stephen, and Victor Menaldo. 2011. "Do Natural Resources Fuel Authoritarianis: A Reappraisal of the Resource Curse." *American Political Science Review* 105 (1): 1–26.

Houle, Christian. 2009. "Inequality and Democracy." *World Politics* 61 (4): 589–622.

Inglehart, Ronald, and Christian Welzel. 2005. *Modernization, Cultural Change and Democracy: The Human Development Sequence.* New York: Cambridge University Press.

Kennedy, Ryan. 2010. "The Contradiction of Modernization: A Conditional Model of Endogenous Democratization." *Journal of Politics* 72 (3): 785–798.

Limongi, Fernando, and Adam Przeworski. 1997. "Modernization: Theories and Facts." *World politics* 49 (2): 155–183.

Moore, Barrington. 1966. *Social origins of dictatorship and democracy: Lord and peasant in the making of the modern world.* Beacon Press: Boston.

Ross, Michael. 2015. "What Have We Learned About the Resource Curse?" *Annual Review of Political Science* 18:239–259.
