# Supplementary material for: Interpretation and identification of within-unit and cross-sectional variation in panel data models
Source: PLoS One. 2020 Apr 21;15(4):e0231349. doi: 10.1371/journal.pone.0231349 (PMC7173782; doi:10.1371/journal.pone.0231349)
Supplement: S3 Appendix — In this appendix we offer additional analysis of 1-way FE estimators as weighted averages and a derivation of the two-way FE estimator in balanced panels. (PDF) [file pone.0231349.s003.pdf]

S3 Appendix Additional Proofs

Jonathan Kropko
School of Data Science
University of Virginia
jkropko@virginia.edu

Robert Kubinec
Department of the Social Sciences
New York University Abu Dhabi
rmk7@nyu.edu
(corresponding author)

March 8, 2020

1 The one-way FE estimator is a weighted average of coefficient estimates within each case/time point

Proposition 1. Let β_i be the coefficient on x_{it} from the regression $y_{it} = \alpha_i + \beta_i x_{it} + \varepsilon_{it}$ that only uses the observations for which the case is i. Without loss of generality, suppose case i is observed for T_i consecutive time points, where the time index t is labeled such that $t \in \{1, 2, \ldots, T_i\}$. By OLS, the coefficient is estimated by

$$\hat{\beta}_i = \frac{\sum_{t=1}^{T_i}(x_{it} - \bar{x}_i)(y_{it} - \bar{y}_i)}{\sum_{t=1}^{T_i}(x_{it} - \bar{x}_i)^2}. \quad (1)$$

The one-way FE estimator is a weighted average of coefficient estimates within each case:

$$\hat{\beta}_{\text{caseFE}} = \frac{\sum_{i=1}^{N} \omega_i \hat{\beta}_i}{\sum_{i=1}^{N} \omega_i}, \quad (2)$$

where

$$\omega_i = \sum_{t=1}^{T_i}(x_{it} - \bar{x}_i)^2 = T_i \times V(x_{it}). \quad (3)$$

Proof. The one-way FE estimator is

$$\hat{\beta}_{\text{caseFE}} = \frac{\sum_{i=1}^{N}\sum_{t=1}^{T_i}(x_{it} - \bar{x}_i)(y_{it} - \bar{y}_i)}{\sum_{i=1}^{N}\sum_{t=1}^{T_i}(x_{it} - \bar{x}_i)^2}. \quad (4)$$

We can multiply and divide a factor of $\sum_{t=1}^{T_i}(x_{it}-\bar{x}_i)^2$ within the summation across cases in the numerator,

$$\hat{\beta}_{\text{caseFE}} = \frac{\sum_{i=1}^{N}\sum_{t=1}^{T_i}(x_{it} - \bar{x}_i)^2 \frac{\sum_{t=1}^{T_i}(x_{it}-\bar{x}_i)(y_{it}-\bar{y}_i)}{\sum_{t=1}^{T_i}(x_{it}-\bar{x}_i)^2}}{\sum_{i=1}^{N}\sum_{t=1}^{T_i}(x_{it} - \bar{x}_i)^2}, \quad (5)$$

which can be rewritten as

$$\hat{\beta}_{\text{caseFE}} = \frac{\sum_{i=1}^{N} \omega_i \hat{\beta}_i}{\sum_{i=1}^{N} \omega_i} \quad (6)$$

where

$$\omega_i = \sum_{t=1}^{T_i} (x_{it} - \bar{x}_i)^2 = T_i \times V(x_{it}). \quad \blacksquare \tag{7}$$

Similarly, the one-way time FE estimator is equal to a weighted average of coefficients derived from OLS on the data subsetted by time point. This proof follows the preceding proof exactly.

2 Derivation of the Two-Way Fixed Effects Estimator for Balanced Panels

Our objective in this section is to derive a clear formula for the two-way FE estimator in balanced panels. A formula, like the well known formula for case FEs in equation 3 in the main text, sheds light on the behavior of this estimator with regard to how it uses the cross-sectional and time variation in the data. Note that this formula builds on the two-way FE transformation in equation 8 in the main text, which is only valid for balanced panels. Therefore this formula is also only valid in the case of balanced panels.

We begin by proving a number of lemmas that we use in the proof of proposition 2 below.

Lemma 1a. The following equivalence holds in both balanced and unbalanced panels:

$$\sum_{i=1}^{N} \sum_{t=1}^{T_i} (x_{it} - \bar{x}_i)(y_{it} - \bar{y}_i) = \sum_{i=1}^{N} \sum_{t=1}^{T_i} (x_{it} - \bar{x}_i)(y_{it} - \bar{y}) = \sum_{i=1}^{N} \sum_{t=1}^{T_i} (x_{it} - \bar{x})(y_{it} - \bar{y}_i). \tag{8}$$

Proof. We demonstrate that all three expressions are individually equal to $\sum_{i=1}^{N} \sum_{t=1}^{T_i} x_{it} y_{it} - \sum_{i=1}^{N} T_i \bar{x}_i \bar{y}_i$ and are therefore equal to each other:

$$\begin{aligned}
\sum_{i=1}^{N} \sum_{t=1}^{T_i} (x_{it} - \bar{x}_i)(y_{it} - \bar{y}_i) &= \sum_{i=1}^{N} \sum_{t=1}^{T_i} (x_{it} y_{it} - x_{it} \bar{y}_i - \bar{x}_i y_{it} + \bar{x}_i \bar{y}_i) \\
&= \sum_{i=1}^{N} \sum_{t=1}^{T_i} x_{it} y_{it} - \sum_{i=1}^{N} \sum_{t=1}^{T_i} x_{it} \bar{y}_i - \sum_{i=1}^{N} \sum_{t=1}^{T_i} \bar{x}_i y_{it} + \sum_{i=1}^{N} \sum_{t=1}^{T_i} \bar{x}_i \bar{y}_i \\
&= \sum_{i=1}^{N} \sum_{t=1}^{T_i} x_{it} y_{it} - \sum_{i=1}^{N} \bar{y}_i \sum_{t=1}^{T_i} x_{it} - \sum_{i=1}^{N} \bar{x}_i \sum_{t=1}^{T_i} y_{it} + \sum_{i=1}^{N} T_i \bar{x}_i \bar{y}_i \\
&= \sum_{i=1}^{N} \sum_{t=1}^{T_i} x_{it} y_{it} - \sum_{i=1}^{N} \bar{y}_i (T_i \bar{x}_i) - \sum_{i=1}^{N} \bar{x}_i (T_i \bar{y}_i) + \sum_{i=1}^{N} T_i \bar{x}_i \bar{y}_i \\
&= \sum_{i=1}^{N} \sum_{t=1}^{T_i} x_{it} y_{it} - \sum_{i=1}^{N} T_i \bar{x}_i \bar{y}_i. \qquad (9)
\end{aligned}$$

$$\begin{aligned}\sum_{i=1}^{N}\sum_{t=1}^{T_i}(x_{it}-\bar{x}_i)(y_{it}-\bar{y}) &= \sum_{i=1}^{N}\sum_{t=1}^{T_i}(x_{it}y_{it}-x_{it}\bar{y}-\bar{x}_i y_{it}+\bar{x}_i\bar{y}) \\ &= \sum_{i=1}^{N}\sum_{t=1}^{T_i}x_{it}y_{it}-\sum_{i=1}^{N}\sum_{t=1}^{T_i}x_{it}\bar{y}-\sum_{i=1}^{N}\sum_{t=1}^{T_i}\bar{x}_i y_{it}+\sum_{i=1}^{N}\sum_{t=1}^{T_i}\bar{x}_i\bar{y} \\ &= \sum_{i=1}^{N}\sum_{t=1}^{T_i}x_{it}y_{it}-\bar{y}\sum_{i=1}^{N}\sum_{t=1}^{T_i}x_{it}-\sum_{i=1}^{N}\bar{x}_i\sum_{t=1}^{T_i}y_{it}+\bar{y}\sum_{i=1}^{N}T_i\bar{x}_i \\ &= \sum_{i=1}^{N}\sum_{t=1}^{T_i}x_{it}y_{it}-\bar{y}\sum_{i=1}^{N}T_i\bar{x}_i-\sum_{i=1}^{N}\bar{x}_i(T_i\bar{y}_i)+\bar{y}\sum_{i=1}^{N}T_i\bar{x}_i \\ &= \sum_{i=1}^{N}\sum_{t=1}^{T_i}x_{it}y_{it}-\sum_{i=1}^{N}T_i\bar{x}_i\bar{y}_i. \qquad (10)\end{aligned}$$

$$\begin{aligned}\sum_{i=1}^{N}\sum_{t=1}^{T_i}(x_{it}-\bar{x})(y_{it}-\bar{y}_i) &= \sum_{i=1}^{N}\sum_{t=1}^{T_i}(x_{it}y_{it}-x_{it}\bar{y}_i-\bar{x}y_{it}+\bar{x}\bar{y}_i) \\ &= \sum_{i=1}^{N}\sum_{t=1}^{T_i}x_{it}y_{it}-\sum_{i=1}^{N}\sum_{t=1}^{T_i}x_{it}\bar{y}_i-\sum_{i=1}^{N}\sum_{t=1}^{T_i}\bar{x}y_{it}+\sum_{i=1}^{N}\sum_{t=1}^{T_i}\bar{x}\bar{y}_i \\ &= \sum_{i=1}^{N}\sum_{t=1}^{T_i}x_{it}y_{it}-\sum_{i=1}^{N}\bar{y}_i\sum_{t=1}^{T_i}x_{it}-\bar{x}\sum_{i=1}^{N}\sum_{t=1}^{T_i}y_{it}+\bar{x}\sum_{i=1}^{N}T_i\bar{y}_i \\ &= \sum_{i=1}^{N}\sum_{t=1}^{T_i}x_{it}y_{it}-\sum_{i=1}^{N}\bar{y}_i(T_i\bar{x}_i)-\bar{x}\sum_{i=1}^{N}T_i\bar{y}_i+\bar{x}\sum_{i=1}^{N}T_i\bar{y}_i \\ &= \sum_{i=1}^{N}\sum_{t=1}^{T_i}x_{it}y_{it}-\sum_{i=1}^{N}T_i\bar{x}_i\bar{y}_i. \quad \blacksquare \qquad (11)\end{aligned}$$

Lemma 1b. The following equivalence holds in both balanced and unbalanced panels:

$$\sum_{i=1}^{N}\sum_{t=1}^{T_i}(x_{it}-\bar{x}_t)(y_{it}-\bar{y}_t)=\sum_{i=1}^{N}\sum_{t=1}^{T_i}(x_{it}-\bar{x}_t)(y_{it}-\bar{y})=\sum_{i=1}^{N}\sum_{t=1}^{T_i}(x_{it}-\bar{x})(y_{it}-\bar{y}_t). \qquad (12)$$

Proof. The proof follows the proof of lemma 1a, substituting $\bar{x}_t$ for $\bar{x}_i$ and $\bar{y}_t$ for $\bar{y}_i$ and rewriting the summation as $\sum_{t=1}^{T}\sum_{i=1}^{N_t}$. $\blacksquare$

Lemma 2a. The following equivalence holds in both balanced and unbalanced panels:

$$\sum_{i=1}^{N}\sum_{t=1}^{T_i}(x_{it}-\bar{x}_i)^2=\sum_{i=1}^{N}\sum_{t=1}^{T_i}(x_{it}-\bar{x}_i)(x_{it}-\bar{x}). \qquad (13)$$

Proof. We demonstrate that both expressions are individually equal to $\sum_{i=1}^{N}\sum_{t=1}^{T_i}x_{it}^2-\sum_{i=1}^{N}T_i\bar{x}_i^2$ and

are therefore equal to each other:

$$\begin{aligned}
\sum_{i=1}^{N}\sum_{t=1}^{T_i}(x_{it}-\bar{x}_i)^2 &= \sum_{i=1}^{N}\sum_{t=1}^{T_i}(x_{it}^2 - 2x_{it}\bar{x}_i + \bar{x}_i^2) \\
&= \sum_{i=1}^{N}\sum_{t=1}^{T_i}x_{it}^2 - 2\sum_{i=1}^{N}\sum_{t=1}^{T_i}x_{it}\bar{x}_i + \sum_{i=1}^{N}\sum_{t=1}^{T_i}\bar{x}_i^2 \\
&= \sum_{i=1}^{N}\sum_{t=1}^{T_i}x_{it}^2 - 2\sum_{i=1}^{N}\bar{x}_i\sum_{t=1}^{T_i}x_{it} + \sum_{i=1}^{N}T_i\bar{x}_i^2 \\
&= \sum_{i=1}^{N}\sum_{t=1}^{T_i}x_{it}^2 - 2\sum_{i=1}^{N}T_i\bar{x}_i^2 + \sum_{i=1}^{N}T_i\bar{x}_i^2 \\
&= \sum_{i=1}^{N}\sum_{t=1}^{T_i}x_{it}^2 - \sum_{i=1}^{N}T_i\bar{x}_i^2. \qquad (14)
\end{aligned}$$

$$\begin{aligned}
\sum_{i=1}^{N}\sum_{t=1}^{T_i}(x_{it}-\bar{x}_i)(x_{it}-\bar{x}) &= \sum_{i=1}^{N}\sum_{t=1}^{T_i}(x_{it}^2 - \bar{x}x_{it} - \bar{x}_i x_{it} + \bar{x}_i\bar{x}) \\
&= \sum_{i=1}^{N}\sum_{t=1}^{T_i}x_{it}^2 - \sum_{i=1}^{N}\sum_{t=1}^{T_i}\bar{x}x_{it} - \sum_{i=1}^{N}\sum_{t=1}^{T_i}\bar{x}_i x_{it} + \sum_{i=1}^{N}\sum_{t=1}^{T_i}\bar{x}_i\bar{x} \\
&= \sum_{i=1}^{N}\sum_{t=1}^{T_i}x_{it}^2 - \bar{x}\sum_{i=1}^{N}\sum_{t=1}^{T_i}x_{it} - \sum_{i=1}^{N}\bar{x}_i\sum_{t=1}^{T_i}x_{it} + \bar{x}\sum_{i=1}^{N}T_i\bar{x}_i \\
&= \sum_{i=1}^{N}\sum_{t=1}^{T_i}x_{it}^2 - \bar{x}\sum_{i=1}^{N}T_i\bar{x}_i - \sum_{i=1}^{N}T_i\bar{x}_i^2 + \bar{x}\sum_{i=1}^{N}T_i\bar{x}_i \\
&= \sum_{i=1}^{N}\sum_{t=1}^{T_i}x_{it}^2 - \sum_{i=1}^{N}T_i\bar{x}_i^2. \quad \blacksquare \qquad (15)
\end{aligned}$$

Lemma 2b. The following equivalence holds in both balanced and unbalanced panels:

$$\sum_{i=1}^{N}\sum_{t=1}^{T_i}(x_{it}-\bar{x}_t)^2 = \sum_{i=1}^{N}\sum_{t=1}^{T_i}(x_{it}-\bar{x}_t)(x_{it}-\bar{x}). \qquad (16)$$

Proof. The proof follows the proof of lemma 2a, substituting $\bar{x}_t$ for $\bar{x}_i$ and rewriting the summation as $\sum_{t=1}^{T}\sum_{i=1}^{N_t}$. $\blacksquare$

Lemma 3. If the panels in the data are balanced, then

$$\sum_{i=1}^{N}\sum_{t=1}^{T}(x_{it}-\bar{x}_i)(y_{it}-\bar{y}_t) = \sum_{i=1}^{N}\sum_{t=1}^{T}(x_{it}-\bar{x}_t)(y_{it}-\bar{y}_i). \qquad (17)$$

Proof. This proof depends on the fact that

$$\bar{x} = \frac{\sum_{i=1}^{N}\sum_{t=1}^{T}x_{it}}{NT} = \frac{\sum_{i=1}^{N}\bar{x}_i}{N} = \frac{\sum_{t=1}^{T}\bar{x}_t}{T} \qquad (18)$$

if and only if the panels are balanced:

$$\begin{aligned}
\sum_{i=1}^{N}\sum_{t=1}^{T}(x_{it}-\bar{x}_i)(y_{it}-\bar{y}_t) &= \sum_{i=1}^{N}\sum_{t=1}^{T}(x_{it}y_{it}-x_{it}\bar{y}_t-\bar{x}_i y_{it}+\bar{x}_i\bar{y}_t) \\
&= \sum_{i=1}^{N}\sum_{t=1}^{T}x_{it}y_{it}-\sum_{i=1}^{N}\sum_{t=1}^{T}x_{it}\bar{y}_t-\sum_{i=1}^{N}\sum_{t=1}^{T}\bar{x}_i y_{it}+\sum_{i=1}^{N}\sum_{t=1}^{T}\bar{x}_i\bar{y}_t \\
&= \sum_{i=1}^{N}\sum_{t=1}^{T}x_{it}y_{it}-\sum_{t=1}^{T}\bar{y}_t\sum_{i=1}^{N}x_{it}-\sum_{i=1}^{N}\bar{x}_i\sum_{t=1}^{T}y_{it}+\sum_{i=1}^{N}\bar{x}_i\sum_{t=1}^{T}\bar{y}_t \\
&= \sum_{i=1}^{N}\sum_{t=1}^{T}x_{it}y_{it}-N\sum_{t=1}^{T}\bar{x}_t\bar{y}_t-T\sum_{i=1}^{N}\bar{x}_i\bar{y}_i+\sum_{i=1}^{N}\bar{x}_i\sum_{t=1}^{T}\bar{y}_t \\
&= \sum_{i=1}^{N}\sum_{t=1}^{T}x_{it}y_{it}-N\sum_{t=1}^{T}\bar{x}_t\bar{y}_t-T\sum_{i=1}^{N}\bar{x}_i\bar{y}_i+NT\bar{x}\bar{y} \\
&= \sum_{i=1}^{N}\sum_{t=1}^{T}x_{it}y_{it}-\sum_{t=1}^{T}\bar{x}_t\sum_{i=1}^{N}y_{it}-\sum_{i=1}^{N}\sum_{t=1}^{T}x_{it}\bar{y}_i+\sum_{i=1}^{N}\bar{x}_t\sum_{t=1}^{T}\bar{y}_i \\
&= \sum_{i=1}^{N}\sum_{t=1}^{T}(x_{it}y_{it}-\bar{x}_t y_{it}-x_{it}\bar{y}_i+\bar{x}_t\bar{y}_i) \\
&= \sum_{i=1}^{N}\sum_{t=1}^{T}(x_{it}-\bar{x}_t)(y_{it}-\bar{y}_i). \quad \blacksquare \qquad (19)
\end{aligned}$$

Lemma 4. If the panels in the data are balanced, then

$$\sum_{i=1}^{N}\sum_{t=1}^{T}\Big[(x_{it}-\bar{x}_i)(y_{it}-\bar{y}_i)+(x_{it}-\bar{x}_t)(y_{it}-\bar{y}_t)-(x_{it}-\bar{x}_i)(y_{it}-\bar{y}_t)\Big]=\sum_{i=1}^{N}\sum_{t=1}^{T}(x_{it}-\bar{x})(y_{it}-\bar{y}). \quad (20)$$

Proof.

$$\sum_{i=1}^{N}\sum_{t=1}^{T}\Big[(x_{it}-\bar{x}_i)(y_{it}-\bar{y}_i)+(x_{it}-\bar{x}_t)(y_{it}-\bar{y}_t)-(x_{it}-\bar{x}_i)(y_{it}-\bar{y}_t)\Big]$$

$$=\sum_{i=1}^{N}\sum_{t=1}^{T}\Big[(x_{it}y_{it}-x_{it}\bar{y}_i-\bar{x}_iy_{it}+\bar{x}_i\bar{y}_i)+(x_{it}y_{it}-x_{it}\bar{y}_t-\bar{x}_ty_{it}+\bar{x}_t\bar{y}_t)-(x_{it}y_{it}-x_{it}\bar{y}_t-\bar{x}_iy_{it}+\bar{x}_i\bar{y}_t)\Big]$$

$$=\sum_{i=1}^{N}\sum_{t=1}^{T}\Big[(x_{it}y_{it}+x_{it}y_{it}-x_{it}y_{it})-x_{it}(\bar{y}_i+\bar{y}_t-\bar{y}_t)-(\bar{x}_i+\bar{x}_t-\bar{x}_i)y_{it}+\bar{x}_i\bar{y}_i+\bar{x}_t\bar{y}_t-\bar{x}_i\bar{y}_t\Big]$$

$$=\sum_{i=1}^{N}\sum_{t=1}^{T}\Big[x_{it}y_{it}-x_{it}\bar{y}_i-\bar{x}_ty_{it}+\bar{x}_i\bar{y}_i+\bar{x}_t\bar{y}_t-\bar{x}_i\bar{y}_t\Big]$$

$$=\sum_{i=1}^{N}\sum_{t=1}^{T}x_{it}y_{it}-\sum_{i=1}^{N}\sum_{t=1}^{T}x_{it}\bar{y}_i-\sum_{i=1}^{N}\sum_{t=1}^{T}\bar{x}_ty_{it}+\sum_{i=1}^{N}\sum_{t=1}^{T}\bar{x}_i\bar{y}_i+\sum_{i=1}^{N}\sum_{t=1}^{T}\bar{x}_t\bar{y}_t-\sum_{i=1}^{N}\sum_{t=1}^{T}\bar{x}_i\bar{y}_t$$

$$=\sum_{i=1}^{N}\sum_{t=1}^{T}x_{it}y_{it}-T\sum_{i=1}^{N}\bar{x}_i\bar{y}_i-N\sum_{t=1}^{T}\bar{x}_t\bar{y}_t+T\sum_{i=1}^{N}\bar{x}_i\bar{y}_i+N\sum_{t=1}^{T}\bar{x}_t\bar{y}_t-NT\bar{x}\bar{y}$$

$$=\sum_{i=1}^{N}\sum_{t=1}^{T}x_{it}y_{it}-NT\bar{x}\bar{y}$$

$$=\sum_{i=1}^{N}\sum_{t=1}^{T}x_{it}y_{it}-NT\bar{x}\bar{y}-NT\bar{x}\bar{y}+NT\bar{x}\bar{y}$$

$$=\sum_{i=1}^{N}\sum_{t=1}^{T}x_{it}y_{it}-\sum_{i=1}^{N}\sum_{t=1}^{T}x_{it}\bar{y}-\sum_{i=1}^{N}\sum_{t=1}^{T}\bar{x}y_{it}+\sum_{i=1}^{N}\sum_{t=1}^{T}\bar{x}\bar{y}$$

$$=\sum_{i=1}^{N}\sum_{t=1}^{T}(x_{it}y_{it}-x_{it}\bar{y}-\bar{x}y_{it}+\bar{x}\bar{y})$$

$$=\sum_{i=1}^{N}\sum_{t=1}^{T}(x_{it}-\bar{x})(y_{it}-\bar{y}). \quad \blacksquare \tag{21}$$

Lemma 5. If the panels in the data are balanced, then

$$\sum_{i=1}^{N}\sum_{t=1}^{T}\Big[(x_{it}-\bar{x}_i)^2+(x_{it}-\bar{x}_t)^2-(x_{it}-\bar{x}_i)(x_{it}-\bar{x}_t)\Big]=\sum_{i=1}^{N}\sum_{t=1}^{T}(x_{it}-\bar{x})^2. \tag{22}$$

Proof.

$$
\begin{aligned}
&\sum_{i=1}^{N}\sum_{t=1}^{T}\Big[(x_{it}-\bar{x}_i)^2+(x_{it}-\bar{x}_t)^2-(x_{it}-\bar{x}_i)(x_{it}-\bar{x}_t)\Big] \\
&=\sum_{i=1}^{N}\sum_{t=1}^{T}\Big[(x_{it}^2-2x_{it}\bar{x}_i+\bar{x}_i^2)+(x_{it}^2-2x_{it}\bar{x}_t+\bar{x}_t^2)-(x_{it}^2-x_{it}\bar{x}_t-x_{it}\bar{x}_i+\bar{x}_i\bar{x}_t)\Big] \\
&=\sum_{i=1}^{N}\sum_{t=1}^{T}\Big[(x_{it}^2+x_{it}^2-x_{it}^2)-x_{it}(2\bar{x}_i+2\bar{x}_t-\bar{x}_i-\bar{x}_t)+\bar{x}_i^2+\bar{x}_t^2-\bar{x}_i\bar{x}_t\Big] \\
&=\sum_{i=1}^{N}\sum_{t=1}^{T}\Big[x_{it}^2-x_{it}\bar{x}_i+x_{it}\bar{x}_t+\bar{x}_i^2+\bar{x}_t^2-\bar{x}_i\bar{x}_t\Big] \\
&=\sum_{i=1}^{N}\sum_{t=1}^{T}x_{it}^2-\sum_{i=1}^{N}\sum_{t=1}^{T}x_{it}\bar{x}_i+\sum_{i=1}^{N}\sum_{t=1}^{T}x_{it}\bar{x}_t+\sum_{i=1}^{N}\sum_{t=1}^{T}\bar{x}_i^2+\sum_{i=1}^{N}\sum_{t=1}^{T}\bar{x}_t^2-\sum_{i=1}^{N}\sum_{t=1}^{T}\bar{x}_i\bar{x}_t \\
&=\sum_{i=1}^{N}\sum_{t=1}^{T}x_{it}^2-T\sum_{i=1}^{N}\bar{x}_i^2+N\sum_{t=1}^{T}\bar{x}_t^2+T\sum_{i=1}^{N}\bar{x}_i^2+N\sum_{t=1}^{T}\bar{x}_t^2-NT\bar{x}^2 \\
&=\sum_{i=1}^{N}\sum_{t=1}^{T}x_{it}^2-NT\bar{x}^2 \\
&=\sum_{i=1}^{N}\sum_{t=1}^{T}x_{it}^2-NT\bar{x}^2+NT\bar{x}^2-NT\bar{x}^2 \\
&=\sum_{i=1}^{N}\sum_{t=1}^{T}x_{it}^2-2\sum_{i=1}^{N}\sum_{t=1}^{T}x_{it}\bar{x}+\sum_{i=1}^{N}\sum_{t=1}^{T}\bar{x}^2 \\
&=\sum_{i=1}^{N}\sum_{t=1}^{T}(x_{it}^2-2x_{it}\bar{x}+\bar{x}^2) \\
&=\sum_{i=1}^{N}\sum_{t=1}^{T}(x_{it}-\bar{x})^2. \quad \blacksquare
\end{aligned}
\tag{23}
$$

Proposition 2. If the panels in the data are balanced, then the two-way FE estimator is given by

$$\hat{\beta}_{TW}=\frac{\sum_{i=1}^{N}\sum_{t=1}^{T}(x_{it}-\bar{x}_i)(y_{it}-\bar{y}_t)}{\sum_{i=1}^{N}\sum_{t=1}^{T}(x_{it}-\bar{x}_i)(x_{it}-\bar{x}_t)}, \tag{24}$$

or equivalently as

$$\hat{\beta}_{TW}=\frac{\sum_{i=1}^{N}\sum_{t=1}^{T}(x_{it}-\bar{x}_t)(y_{it}-\bar{y}_i)}{\sum_{i=1}^{N}\sum_{t=1}^{T}(x_{it}-\bar{x}_i)(x_{it}-\bar{x}_t)}. \tag{25}$$

Proof. The two-way fixed effects estimator is the OLS estimator applied to the following model,

$$y_{it}^*=\alpha_{TW}+\beta_{TW}x_{it}^*+\varepsilon_{it}, \tag{26}$$

where $y^*_{it} = y_{it} - \bar{y}_i - \bar{y}_t + \bar{y}$ and $x^*_{it} = x_{it} - \bar{x}_i - \bar{x}_t + \bar{x}$. By OLS, the coefficient in equation 26 is given by

$$\begin{aligned}
\hat{\beta}_{TW} &= \frac{\sum_{i=1}^{N}\sum_{t=1}^{T_i}\Big(x_{it} - \bar{x}_i - \bar{x}_t + \bar{x}\Big)\Big(y_{it} - \bar{y}_i - \bar{y}_t + \bar{y}\Big)}{\sum_{i=1}^{N}\sum_{t=1}^{T_i}\Big(x_{it} - \bar{x}_i - \bar{x}_t + \bar{x}\Big)^2} \\
&= \frac{\sum_{i=1}^{N}\sum_{t=1}^{T_i}\Big(x_{it} + x_{it} - x_{it} - \bar{x}_i - \bar{x}_t + \bar{x}\Big)\Big(y_{it} + y_{it} - y_{it} - \bar{y}_i - \bar{y}_t + \bar{y}\Big)}{\sum_{i=1}^{N}\sum_{t=1}^{T_i}\Big(x_{it} + x_{it} - x_{it} - \bar{x}_i - \bar{x}_t + \bar{x}\Big)^2} \\
&= \frac{\sum_{i=1}^{N}\sum_{t=1}^{T_i}\Big[(x_{it} - \bar{x}_i) + (x_{it} - \bar{x}_t) - (x_{it} - \bar{x})\Big]\Big[(y_{it} - \bar{y}_i) + (y_{it} - \bar{y}_t) - (y_{it} - \bar{y})\Big]}{\sum_{i=1}^{N}\sum_{t=1}^{T_i}\Big[(x_{it} - \bar{x}_i) + (x_{it} - \bar{x}_t) - (x_{it} - \bar{x})\Big]^2}, \\
&= \frac{\sum_{i=1}^{N}\sum_{t=1}^{T_i} A_{it}}{\sum_{i=1}^{N}\sum_{t=1}^{T_i} B_{it}}, \qquad (27)
\end{aligned}$$

where

$$\begin{aligned}
A_{it} &= (x_{it} - \bar{x}_i)(y_{it} - \bar{y}_i) + (x_{it} - \bar{x}_i)(y_{it} - \bar{y}_t) - (x_{it} - \bar{x}_i)(y_{it} - \bar{y}) \\
&\quad + (x_{it} - \bar{x}_t)(y_{it} - \bar{y}_i) + (x_{it} - \bar{x}_t)(y_{it} - \bar{y}_t) - (x_{it} - \bar{x}_t)(y_{it} - \bar{y}) \\
&\quad - (x_{it} - \bar{x})(y_{it} - \bar{y}_i) - (x_{it} - \bar{x})(y_{it} - \bar{y}_t) + (x_{it} - \bar{x})(y_{it} - \bar{y}), \qquad (28)
\end{aligned}$$

and

$$\begin{aligned}
B_{it} &= (x_{it} - \bar{x}_i)^2 + (x_{it} - \bar{x}_t)^2 + (x_{it} - \bar{x})^2 \\
&\quad + 2(x_{it} - \bar{x}_i)(x_{it} - \bar{x}_t) - 2(x_{it} - \bar{x}_i)(x_{it} - \bar{x}) - 2(x_{it} - \bar{x}_t)(x_{it} - \bar{x}). \qquad (29)
\end{aligned}$$

From lemmas 1a, 1b, 2a, and 2b, A_{it} and B_{it} reduce to

$$\begin{aligned}
A_{it} &= (x_{it} - \bar{x})(y_{it} - \bar{y}) - (x_{it} - \bar{x}_i)(y_{it} - \bar{y}_i) - (x_{it} - \bar{x}_t)(y_{it} - \bar{y}_t) \\
&\quad + (x_{it} - \bar{x}_i)(y_{it} - \bar{y}_t) + (x_{it} - \bar{x}_t)(y_{it} - \bar{y}_i), \qquad (30)
\end{aligned}$$

and

$$B_{it} = (x_{it} - \bar{x})^2 - (x_{it} - \bar{x}_i)^2 - (x_{it} - \bar{x}_t)^2 + 2(x_{it} - \bar{x}_i)(x_{it} - \bar{x}_t). \qquad (31)$$

By lemma 3, A_{it} simplifies to

$$A_{it} = (x_{it} - \bar{x})(y_{it} - \bar{y}) - (x_{it} - \bar{x}_i)(y_{it} - \bar{y}_i) - (x_{it} - \bar{x}_t)(y_{it} - \bar{y}_t) + 2(x_{it} - \bar{x}_i)(y_{it} - \bar{y}_t), \qquad (32)$$

or equivalently to

$$A_{it} = (x_{it} - \bar{x})(y_{it} - \bar{y}) - (x_{it} - \bar{x}_i)(y_{it} - \bar{y}_i) - (x_{it} - \bar{x}_t)(y_{it} - \bar{y}_t) + 2(x_{it} - \bar{x}_t)(y_{it} - \bar{y}_i). \qquad (33)$$

By lemma 4, A_{it} further simplifies to

$$
\begin{aligned}
A_{it} = &\Big[(x_{it} - \bar{x}_i)(y_{it} - \bar{y}_i) + (x_{it} - \bar{x}_t)(y_{it} - \bar{y}_t) - (x_{it} - \bar{x}_i)(y_{it} - \bar{y}_t)\Big] \\
&- (x_{it} - \bar{x}_i)(y_{it} - \bar{y}_i) - (x_{it} - \bar{x}_t)(y_{it} - \bar{y}_t) + 2(x_{it} - \bar{x}_i)(y_{it} - \bar{y}_t) \\
&= (x_{it} - \bar{x}_i)(y_{it} - \bar{y}_t),
\end{aligned}
\tag{34}
$$

or equivalently to $(x_{it} - \bar{x}_t)(y_{it} - \bar{y}_i)$ by lemma 3. Applying lemma 5, B_{it} reduces as follows:

$$
\begin{aligned}
B_{it} = &\Big[(x_{it} - \bar{x}_i)^2 + (x_{it} - \bar{y}_t)^2 - (x_{it} - \bar{x}_i)(x_{it} - \bar{x}_t)\Big] \\
&- (x_{it} - \bar{x}_i)^2 - (x_{it} - \bar{x}_t)^2 + 2(x_{it} - \bar{x}_i)(x_{it} - \bar{x}_t) \\
&= (x_{it} - \bar{x}_i)(x_{it} - \bar{x}_t).
\end{aligned}
\tag{35}
$$

Therefore, the two-way fixed effect estimator is given by

$$
\hat{\beta}_{TW} = \frac{\sum_{i=1}^{N} \sum_{t=1}^{T} (x_{it} - \bar{x}_i)(y_{it} - \bar{y}_t)}{\sum_{i=1}^{N} \sum_{t=1}^{T} (x_{it} - \bar{x}_i)(x_{it} - \bar{x}_t)}, \tag{36}
$$

or equivalently as

$$
\hat{\beta}_{TW} = \frac{\sum_{i=1}^{N} \sum_{t=1}^{T} (x_{it} - \bar{x}_t)(y_{it} - \bar{y}_i)}{\sum_{i=1}^{N} \sum_{t=1}^{T} (x_{it} - \bar{x}_i)(x_{it} - \bar{x}_t)}. \quad \blacksquare \tag{37}
$$
